# Trained Memory of Uterine Macrophages Improves Subsequent Pregnancy Outcomes

**DOI:** 10.1002/advs.74889

**Published:** 2026-03-28

**Authors:** Jing Wang, Ning Lu, Xin‐Xiu Lin, Xu‐Hui Fang, Yi‐Hui Li, Qing‐Peng Luo, Yu‐Ting Peng, Gil Mor, Ai‐Hua Liao

**Affiliations:** ^1^ Institute of Reproductive Health Center for Reproductive Medicine Tongji Medical College Huazhong University of Science and Technology Wuhan P.R. China; ^2^ C.S. Mott Center for Human Growth and Development Wayne State University School of Medicine Detroit Michigan USA

**Keywords:** decidual macrophages, innate immune memory, leukocyte immunoglobulin‐like receptor subfamily B, maternal‐fetal interface, paired immunoglobulin‐like receptor B

## Abstract

A previously successful pregnancy promotes the fitness of subsequent pregnancies, of which the pregnancy‐induced memory is ascribed exclusively to adaptive lymphocytes. However, whether macrophages at maternal‐fetal interface acquire pregnancy‐specific immune memory remains unclear. Using human decidual samples and complementary mouse models, we identify human leukocyte immunoglobulin‐like receptor subfamily B3^+^ (LILRB3^+^) and murine paired immunoglobulin‐like receptor B^+^ (PIR‐B^+^) macrophages as a uterine memory subset, which expands progressively with gravidity and gestational age, and exhibits paternal specific immune memory. In both species, these cells exhibited hallmarks of pregnancy‐induced trained tolerance, including elevated IL‐10, TGF‐β, and CD206, together with reduced CD80/CD86 expression and suppression of pro‐inflammatory cytokines. Mechanistically, PIR‐B‐SHP signaling drives macrophage metabolic reprogramming to oxidative phosphorylation/fatty acid oxidation for the formation of memory. Moreover, adoptive transfer of PIR‐B^+^ uterine macrophages into abortion‐prone mouse models significantly improves pregnancy outcomes, highlighting their therapeutic potential. Together, our findings uncover a previously unrecognized form of decidual macrophage trained memory in an antigen‐specific manner, which opens avenues for therapeutic strategies aimed at preventing or reducing recurrent pregnancy loss.

## Graphical abstract

This study identifies that pregnancy imprints a durable, pregnancy‐specific form of trained immune memory in uterine macrophages, marked by the emergence of LILRB3^+^/PIR‐B^+^ cells that expand across gestations, acquire a tolerogenic and metabolically rewired phenotype, and actively protect against inflammatory pregnancy loss in mice. These findings broaden the scope of trained immunity and reveal their potential roles in reproductive success.

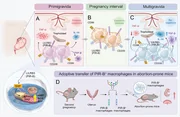

## 1 Introduction

Pregnancy represents one of the most extraordinary immunological paradoxes in biology [1]. The maternal immune system must simultaneously defend against infection and tolerate the allogeneic fetus that expresses paternal antigens [2, 3]. This balance is achieved not through global immune suppression but through an active process of immune adaptation that reshapes both systemic and local immune responses [4, 5]. The uterine lining, or decidua, provides the central arena for this dialogue. Here, specialized populations of decidual macrophages (DMs), uterine natural killer (uNK) cells, dendritic cells, and regulatory T cells (Tregs) coordinate implantation, placental development, and maintenance of tolerance. Among these, macrophages are uniquely versatile: they participate in tissue remodeling [6], angiogenesis [7], clearance of apoptotic cells [8], and modulation of trophoblast invasion [9]. Their remarkable plasticity allows them to oscillate between pro‐inflammatory and tolerogenic states across gestation. After delivery, however, the placenta and most pregnancy‐associated leukocytes are shed, raising a fundamental question: how these cells adapt across multiple pregnancies, and whether prior gestations leave a durable imprint on uterine macrophages, remain largely unknown.

Clinical and epidemiologic observations suggest that the maternal immune system “remembers” previous pregnancies [10, 11]. Multiparous women generally exhibit improved placental function, larger neonatal weights, and reduced risks of preeclampsia (PE) and spontaneous abortion compared with primigravid women [12, 13, 14]. These benefits, however, are often lost when conception occurs with a new paternal partner—an effect sometimes termed the “same‐father advantage” [15, 16]. Experimental studies have mirrored these findings: in mice, second pregnancies display enhanced placental vascularization and fetal growth, whereas antigen‐switch mating abolishes this benefit [17]. These patterns imply that prior exposure to paternal antigens induces a form of antigen‐specific tolerance.

Traditionally, pregnancy‐induced memory has been ascribed exclusively to adaptive lymphocytes, particularly T cells, much less is known about innate immune memory at the maternal‐fetal interface. Specifically, antigen‐specific memory Tregs persist after pregnancy and promote fetal tolerance in subsequent gestations [18], and a subset of memory Tregs derived from Th17 cells has been identified via surface markers CXCR4 and CD274 [17]. Fetal‐specific CD8^+^ T cells are also detectable postpartum but exhibit reduced effector function in subsequent pregnancies, thereby mitigating anti‐fetal immunity [19]. Our previous work further revealed that pregnancy‐induced alterations in immune cell composition persist in peripheral blood for up to one year postpartum [20].

However, the concept of ‘trained immunity’ has redefined this paradigm, demonstrating that innate cells can acquire long‐lasting functional reprogramming after transient stimulation [21, 22, 23]. This training can produce either enhanced inflammatory responsiveness or, conversely, a state of tolerance depending on the nature of the initial signal. β‐glucan or BCG vaccination generates pro‐inflammatory training through activation of mTOR–HIF‐1α pathways and increased glycolysis [24], whereas exposure to apoptotic cells or chronic endotoxin induces a “trained tolerance” characterized by anti‐inflammatory cytokine profiles and reliance on oxidative phosphorylation (OXPHOS) [25]. These processes are underpinned by epigenetic remodeling and metabolic rewiring that persist beyond cell turnover.

Tissue‐resident macrophages are especially poised to develop such memory. As sentinels within organs, they integrate recurrent environmental signals—microbial, hormonal, metabolic, or antigenic—and convert them into enduring transcriptional and metabolic programs. In the liver, Kupffer cells retain memory of PEGylated nanoemulsions [26]; in the lung, alveolar macrophages remember allergen exposure [27, 28]; in the brain, microglia maintain long‐term epigenetic scars following inflammation [29]. Whether uterine macrophages undergo similar imprinting following pregnancy—a cyclical, antigenically complex, and metabolically demanding state—has remained unexplored. Interestingly, a landmark study in mice demonstrated that macrophages can develop alloantigen‐specific memory, mediated by paired immunoglobulin‐like receptors (PIR), with PIR‐B promoting tolerogenic immune memory [30]. Its human functional and structural homolog, leukocyte Ig‐like receptor subfamily B (LILRB), is a kind of inhibitory receptors widely expressed in macrophages and exhibits structural and functional conservation with murine PIR‐B [31]. Importantly, LILRB1 has been identified as a marker of memory decidual NK cells, and these subsets remember the first pregnancy and better assist proper placentation in subsequent pregnancies by producing IFN‐γ and VEGF‐α [32]. These findings raise the compelling possibility that LILRB receptors might also mediate fetal‐specific memory in macrophages.

Here, we sought to determine whether pregnancy induces a durable, antigen‐dependent macrophage memory program at the maternal–fetal interface. Using human decidual samples and complementary mouse models, we identified LILRB3^+^ (human) and PIR‐B^+^ (mouse) macrophages as a conserved macrophage subset, which expand progressively with gravidity and gestational age, and exhibit trained tolerance properties. Transcriptomic/metabolic analyses showed their functional phenotype depends on the PIR‐B–SHP axis, essential for memory establishment. Adoptive transfer of PIR‐B^+^ macrophages from secondary pregnancies rescued fetal loss and restored placental vasculature in lipopolysaccharide (LPS)‐induced abortion‐prone mice, indicating that these macrophages confer transferrable immune resilience. Together, our findings uncover a previously unrecognized form of decidual macrophage memory (induced by prior pregnancy, and maintained via metabolic reprogramming), which broadens the scope of trained immunity and reveals their roles in reproductive success.

## 2 Results

### 2.1 LILRB3^+^ DMs Expand with Gravidity and Gestational Progression

**FIGURE 1:**
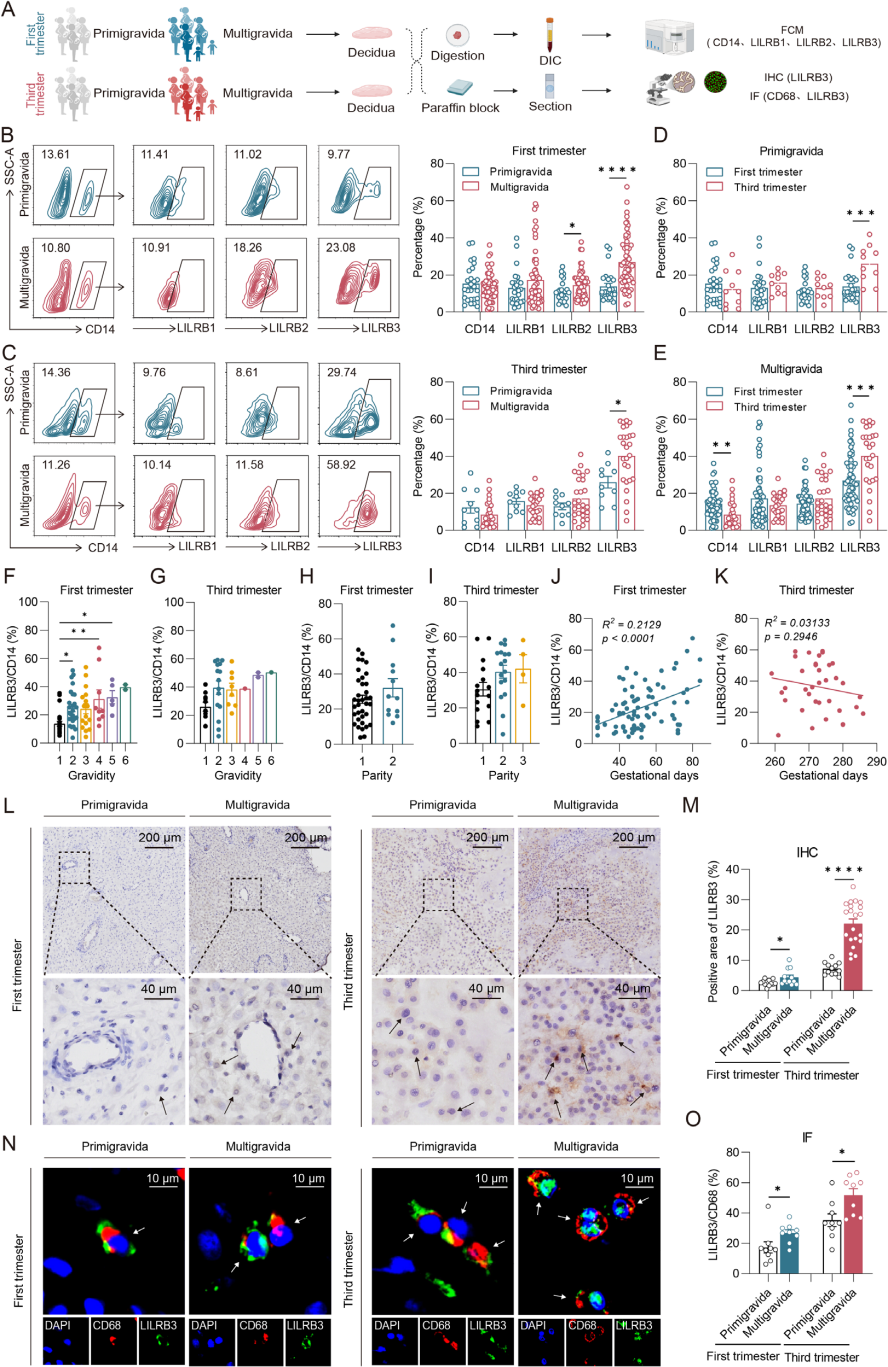
Leukocyte immunoglobulin‐like receptor subfamily B3 (LILRB3) is upregulated on decidual macrophages (DMs) in multigravid women. (A) Schematic workflow of decidual tissue collection and processing. (B and C) Flow cytometric (FCM) analysis of LILRB1–3 expression on DMs in (B) first‐trimester (Primigravid, n = 26; Multigravid, n = 58) and (C) third‐trimester (Primigravid, n = 10; Multigravid, n = 27) pregnancies. (D and E) FCM analysis of LILRB1–3 expression on DMs in (D) primigravid (First‐trimester, n = 26; Third‐trimester, n = 10) and (E) multigravid (First‐trimester, n = 58; Third‐trimester, n = 27) women. (F–K) Correlation between the proportion of LILRB3^+^ DMs and gravidity (F, G), parity (H, I), and gestational days (J, K) in first‐trimester and third‐trimester pregnancies. (L and M) Representative immunohistochemical (IHC) staining of LILRB3 (L) in decidual tissues and its quantitative (M) analysis. The proportion of positive cells by IHC was determined by calculating the ratio of positively stained regions to the total visual field area across randomly selected high‐power fields per tissue section. Image analysis was performed using ImageJ. Scale bars, 200 µm (overview) and 40 µm (inset). (N and O) Representative immunofluorescence (IF) images of decidual tissues co‐stained for the macrophage marker CD68 (red) and LILRB3 (green) (N) and its quantitative (O) analysis. Nuclei are counterstained with DAPI (blue). Scale bar, 10 µm. Data in B–I, M, O are presented as mean ± SEM. Pearson correlation analysis was used in J and K. Statistical differences were determined by unpaired two‐tailed t‐test in B‐E, H, M, O and by one‐way ANOVA in F, G, I; ^*^ *p* < 0.05, ^**^ *p* < 0.01, ^***^ *p* < 0.001, ^****^ *p* < 0.0001.

To explore whether DMs acquire a form of tissue‐resident memory induced by prior pregnancies, we analyzed first‐ and third‐trimester decidual samples from primigravid and multigravid women (Figure 1A). Flow cytometric profiling of inhibitory receptors revealed that LILRB3 was selectively upregulated on DMs from multigravida women during the first trimester, whereas LILRB1 and LILRB2 showed modest or no change (Figure 1B). This LILRB3‐dominant phenotype persisted into the third trimester (Figure 1C), suggesting a stable adaptation of the macrophage compartment across pregnancies.

Across gestation, LILRB3 expression increased significantly from the first to the third trimester in both primigravid and multigravida groups (Figure 1D,E). Quantitative analyses further demonstrated that the frequency of LILRB3^+^ DMs correlated with gravidity and gestational age (Figure 1F–K), but not with parity (Figure 1H,I), indicating that macrophage “memory” accumulation is linked to repeated pregnancies rather than delivery history. In addition, no statistically significant correlation was identified between maternal ages and the frequencies of LILRB3^+^ DMs in both the first and third trimesters (Figure S1). Immunohistochemistry and immunofluorescence confirmed the enrichment of LILRB3^+^ macrophages within the decidua (Figure 1L–O), supporting the cytometric data. Collectively, these findings identify LILRB3^+^ DMs as a gravidity‐dependent macrophage subset that expands progressively during gestation, consistent with the emergence of a pregnancy‐induced tissue memory program within the maternal–fetal interface.

### 2.2 PIR‐B^+^ Uterine Macrophages Expand in a Paternal Antigen–Specific Manner during Second Pregnancy in Mice

**FIGURE 2:**
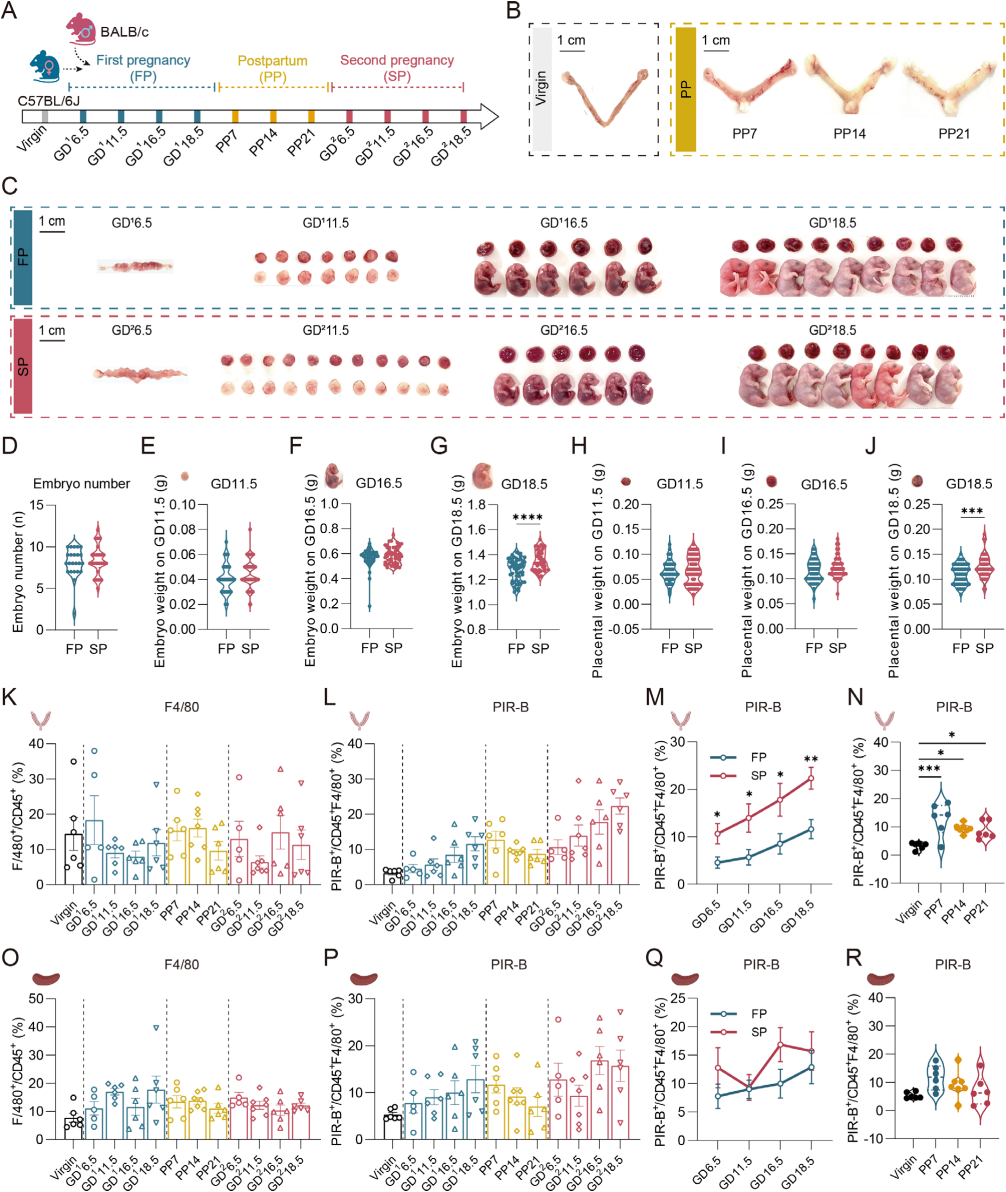
Paired immunoglobulin‐like receptor B (PIR‐B) is upregulated on uterine macrophages in secondary pregnancies. (A) Schematic of the experimental mouse models: Virgin, first pregnancy (FP), postpartum (PP), second pregnancy (SP). (B) Representative uterine images from Virgin, PP7, PP14, and PP21 mice. Scale bar, 1 cm. (C) Morphology of fetuses and placentas at gestational day (GD) 6.5, 11.5, 16.5, and 18.5 in FP and SP. Scale bar, 1 cm. (D) Number of fetuses in FP and SP. (E–G) Fetal weights in FP and SP at (E) GD11.5, (F) GD16.5, and (G) GD18.5. (H–J) Placental weights in FP and SP at (H) GD11.5, (I) GD16.5, and (J) GD18.5. (K) Proportion of uterine macrophages during FP and SP. (L) PIR‐B expression on uterine macrophages during FP and SP. (M) Comparison of PIR‐B levels on uterine macrophages between FP and SP at GD6.5, 11.5, 16.5, and 18.5. (N) PIR‐B levels on uterine macrophages in Virgin, PP7, PP14, and PP21 mice. (O) Proportion of splenic macrophages during FP and SP. (P) PIR‐B expression on splenic macrophages during FP and SP. (Q) Comparison of PIR‐B levels on splenic macrophages between FP and SP at GD6.5, 11.5, 16.5, and 18.5. (R) PIR‐B levels on splenic macrophages in Virgin, PP7, PP14, and PP21 mice. Data in D–J, K, L, N, O, P, R are presented as mean ± SEM. Statistical differences were determined by unpaired two‐tailed *t*‐test in D‐J, M, Q and by one‐way ANOVA in N, R; ^*^ *p* < 0.05, ^**^ *p* < 0.01, ^***^ *p* < 0.001, ^****^ *p* < 0.0001.

To determine whether uterine macrophages develop pregnancy‐induced immune memory, we used mouse models in which C57BL/6J females were mated with BALB/c males for first and second pregnancies (Figure 2A). Compared with virgin mice, postpartum uteri exhibited sustained enlargement through postpartum day 21 (PP21) (Figure 2B). Although fetal numbers were comparable between first and second pregnancies (Figure 2C,D), both placental and fetal weights increased significantly during late‐stage second pregnancies (Figure 2E–J), indicating improved gestational performance upon repeated allogeneic exposure.

Flow cytometric analysis revealed no difference in total macrophage proportions between pregnancies (Figure 2K), but a progressive accumulation of PIR‐B^+^ uterine macrophages was observed after the first pregnancy (Figure 2L), with elevated frequencies maintained postpartum (Figure 2N). This expansion was further amplified during the second pregnancy relative to matched first‐pregnancy timepoints (Figure 2M). In contrast, splenic macrophages showed low baseline PIR‐B expression that remained unchanged across pregnancies (Figure 2O–R), suggesting that PIR‐B^+^ macrophage expansion is tissue‐restricted to the uterus.

**FIGURE 3:**
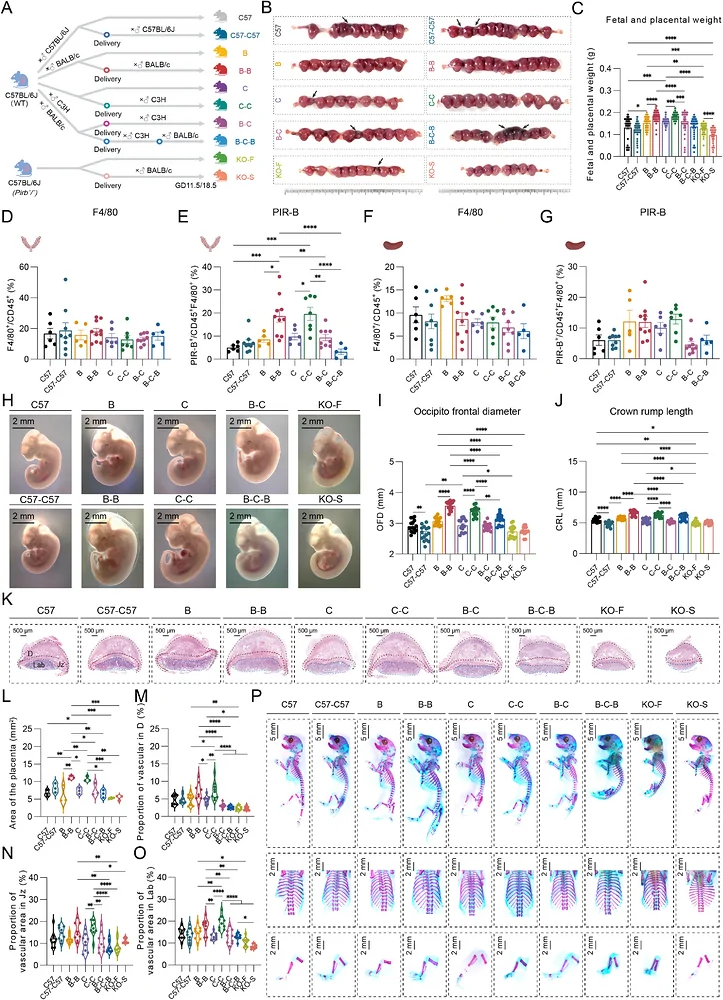
Pregnancy‐induced PIR‐B^+^ uterine macrophages exhibit paternity specificity. (A) Schematic of the syngeneic and allogeneic pregnancy models and the corresponding group nomenclature. C57: C57BL/6J♀×C57BL/6J♂ (first pregnancy); C57‐C57: C57BL/6J♀× C57BL/6J♂ (first and second pregnancies); B: C57BL/6J♀× BALB/c♂ (first pregnancy); B‐B: C57BL/6J♀×BALB/c♂ (first and second pregnancies); C: C57BL/6J♀×C3H♂ (first pregnancy); C‐C: C57BL/6J♀×C3H♂ (first and second pregnancies); B‐C: C57BL/6J♀×BALB/c♂ (first pregnancy), then C57BL/6J♀× C3H♂ (second pregnancy); B‐C‐B: C57BL/6J♀×BALB/c♂ (first pregnancy), then C57BL/6J♀×C3H♂ (second pregnancy), finally C57BL/6J♀× BALB/c♂(third pregnancy); KO‐F: *Pirb* ^−^/^−^ C57BL/6J♀×BALB/c♂ (first pregnancy); KO‐S: *Pirb* ^−^/^−^ C57BL/6J♀×BALB/c♂ (first and second pregnancies). (B) Uterine morphology at GD11.5. (C) Fetal‐placental weights at GD11.5. (D) Proportion of uterine macrophages at GD11.5. (E) PIR‐B expression on uterine macrophages at GD11.5. (F) Proportion of splenic macrophages at GD11.5. (G) PIR‐B expression on splenic macrophages at GD11.5. (H) Representative embryo images at GD11.5. Scale bar, 2 mm. (I and J) Embryonic (I) occipito‐frontal diameter (OFD) and (J) crown‐rump length (CRL) at GD11.5. (K) H&E staining of placental sections at GD11.5. Scale bar, 500 µm. (L) Total placental area at GD 11.5. For each group, one placenta from each of three randomly selected mice was analyzed. (M–O) Vascular area proportion in the (M) decidua (D), (N) junctional zone (Jz), and (O) labyrinth (Lab) at GD11.5. Three random fields of view per region were quantified for vascular area. (P) Representative Alcian blue‐stained skeletons of GD18.5 fetuses. Scale bars, 5 mm (overview), 2 mm (detail). For quantification in (H‐J), three mice per group were randomly selected, and five embryos from each mouse were measured. Data in C–G, I, J, L‐O are presented as mean ± SEM. Statistical differences were determined by one‐way ANOVA in C–G, I, J, L‐O; ^*^ *p* < 0.05, ^**^ *p* < 0.01, ^***^ *p* < 0.001, ^****^ *p* < 0.0001.

Given prior evidence that PIR family receptors mediate macrophage memory responses and the paternal‐lineage specificity of improved reproductive outcomes [30, 33], we hypothesized that PIR‐B^+^ macrophage memory in pregnancy would exhibit paternal antigen dependence. To test this, we compared syngeneic (C57BL/6J × C57BL/6J) and allogeneic (C57BL/6J × BALB/c or C3H) mating models (Figure 3A). In syngeneic second pregnancies, there was no increase in fetal–placental weight (Figure 3B,C and Figure S2A), no expansion of PIR‐B^+^ macrophages in either uterus or spleen (Figure 3D–G), and reduced fetal growth metrics, including occipito–frontal diameter (OFD) and crown–rump length (CRL) (Figure 3H–J). These data indicate that allogeneic stimulation is required to induce macrophage memory and optimize gestational outcomes.

In contrast, repeated exposure to the same paternal strain (B–B or C–C) elicited robust improvements in fetal–placental weight (Figure 3B,C and Figure S2A), expansion of PIR‐B^+^ uterine macrophages (Figure 3D,E), enhanced fetal size (OFD and CRL) (Figure 3H–J), placental vascular remodeling (Figure 3K–O and Figure S2B–F), and advanced neonatal development at gestation day 18.5 (GD18.5) (Figure 3P). Notably, antigen‐switch (B–C) and interrupted‐exposure (B–C–B) mating abolished these effects (Figure 3B–P and Figure S2), confirming paternal antigen specificity.

To directly test PIR‐B's function, *Pirb* ^−^/^−^ females exhibited marked impairments in placental and fetal growth (Figure 3B, C, H–J and Figure S2A), abnormal placental morphology (Figure 3K–O and Figure S2B–F), and delayed neonatal development (Figure 3P), which were further exacerbated in second pregnancies.

Together, these findings establish that PIR‐B^+^ uterine macrophages represent a pregnancy‐induced, paternal antigen–specific memory subset that expands selectively during subsequent allogeneic pregnancies, functioning to enhance placental vascularization and fetal growth across gestations.

### 2.3 LILRB3^+^/PIR‐B^+^ Uterine Macrophages Exhibit Enhanced Immune Tolerance During Subsequent Pregnancies

**FIGURE 4:**
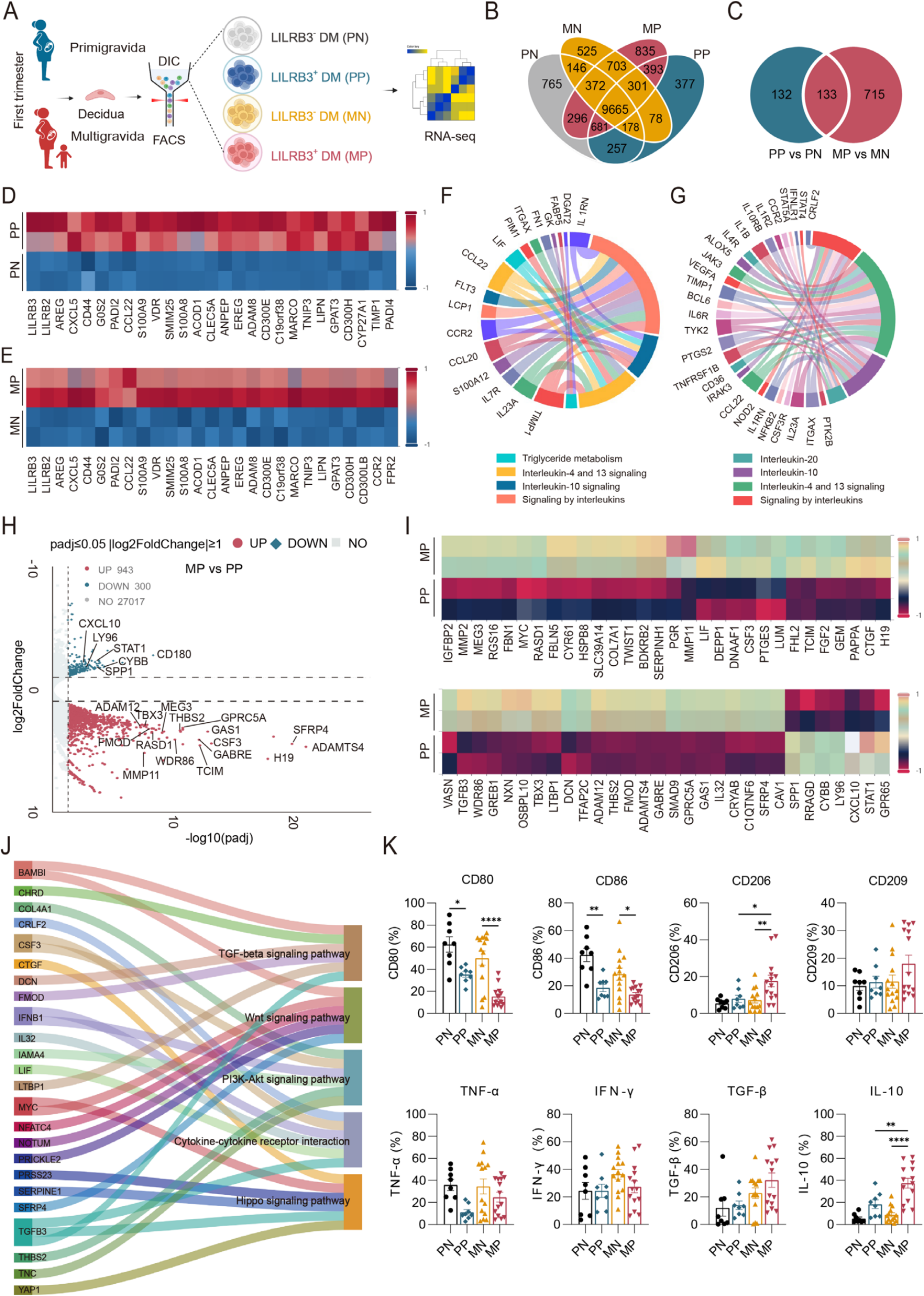
Transcriptomic and functional characteristics of human LILRB3^+^ macrophages. (A) Schematic of FCM sorting and transcriptome sequencing of LILRB3^−^ and LILRB3^+^ macrophages from primary (PN, LILRB3^−^ macrophages; PP, LILRB3^+^ macrophages) and multiple (MN, LILRB3^−^ macrophages; MP, LILRB3^+^ macrophages) pregnancies. (DIC: decidual immune cells). (B) Venn diagram of expressed genes across PN, PP, MN, and MP groups. (C) Venn analysis comparing differentially expressed genes (DEGs) from (PP vs PN) and (MP vs MN). (D) Heatmap of DEGs between PN and PP. (E) Heatmap of DEGs between MN and MP. (F) Enrichment analysis of DEGs in PP vs PN. (G) Enrichment analysis of DEGs in MP vs MN. (H) Volcano plot of DEGs between MP and PP. (I) Heatmap of DEGs between PP and MP. (J) Enrichment analysis of DEGs between MP and PP. (K) M1/M2 marker expression and cytokine production profiles in PN, PP, MN, and MP. Data in K are presented as mean ± SEM. Statistical differences were determined by one‐way ANOVA in K; ^*^ *p* < 0.05, ^**^ *p* < 0.01, ^****^ *p* < 0.0001.

To define the molecular basis of pregnancy‐induced macrophage memory, we profiled human LILRB3^−^/^+^ DMs isolated by flow cytometry with a purity of over 90% from first‐trimester decidua of primigravid (PN, PP) and multigravida (MN, MP) women (Figure 4A). Transcriptomic analysis revealed distinct expression patterns among groups (Figure 4B), with 133 shared differentially expressed genes (DEGs) between first and subsequent pregnancies (PP vs PN; MP vs MN), including LILRB3, LILRB2, and CD44 (Figure 4C). Genes upregulated in both PP and MP were enriched for M2 polarization markers (LILRB3, LILRB2, FPR2), angiogenic factors (EREG, ANPEP, AREG), and pregnancy‐associated regulators (ACOD1, ADAM8, CXCL5) (Figure 4D,E). Pathway enrichment analysis highlighted the activation of IL‐4/IL‐13 and IL‐10 signaling cascades (Figure 4F,G), which might be related to the tolerogenic phenotypes observed in LILRB3^+^ macrophages.

Comparison of multigravida (MP) vs primigravid (PP) pregnancies identified 943 upregulated genes associated with M2 polarization (IGFBP2, THBS2, SPON2), angiogenesis (ADAMTS4, TBX3, CYR61), cell proliferation and metabolism (H19, BCAR1, GABRE, MEG3, IGFBP6), and pregnancy remodeling pathways (CSF3, ADAM12, GREB1, BMP2, MMP2) (Figure 4H,I). Enhanced TGF‐β signaling further suggested an amplified tolerogenic profile in LILRB3^+^ DMs during subsequent pregnancies (Figure 4J).

Consistent with these transcriptomic patterns, flow cytometric validation revealed that first pregnancies (PP) exhibited reduced expression of co‐stimulatory molecules CD80 and CD86 relative to PN, while second pregnancies (MP) showed further reductions in CD80/CD86 and elevated CD206 and IL‐10 compared with MN (Figure 4K). Direct comparison confirmed that MP macrophages expressed higher CD206 and IL‐10 than PP, indicating a progressive acquisition of tolerogenic features across pregnancies.

**FIGURE 5:**
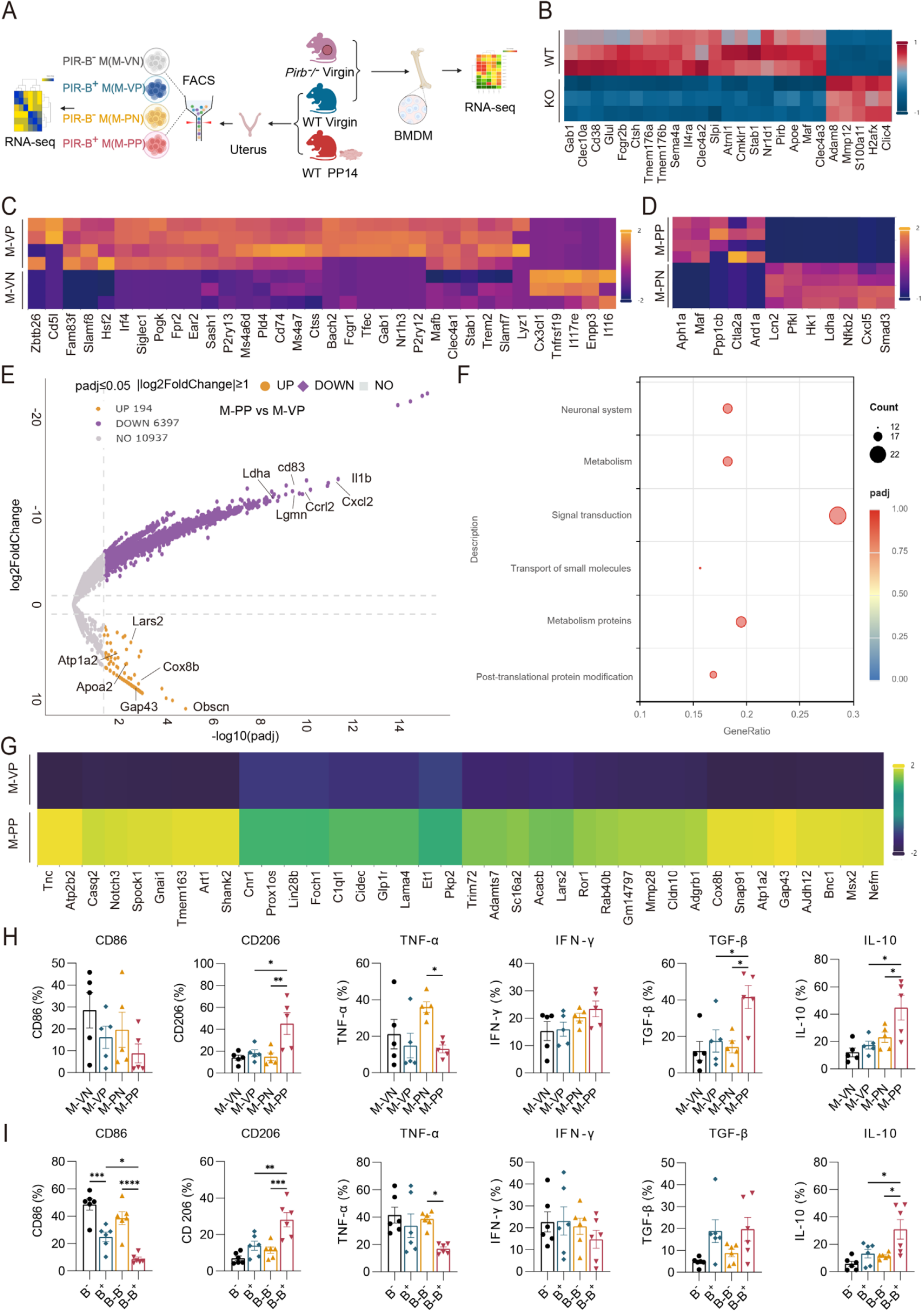
Transcriptomic and functional characteristics of mice uterine PIR‐B^+^ macrophages. (A) Sorting and RNA‐seq of PIR‐B^−^ and PIR‐B^+^ uterine macrophages from virgin (M‐VN: PIR‐B^−^ macrophages, M‐VP: PIR‐B^+^ macrophages) and PP14 (M‐PN: PIR‐B^−^ macrophages, M‐PP: PIR‐B^+^ macrophages) mice (left); workflow for obtaining and sequencing WT vs *Pirb* ^−^/^−^ bone marrow‐derived macrophages (BMDMs) (right). (B) Heatmap of DEGs between WT and Pirb^−^/^−^ BMDMs. (C) Heatmap of DEGs between M‐VP and M‐VN groups. (D) Heatmap of DEGs between M‐PP and M‐PN groups. (E) Volcano plot of DEGs between M‐PP and M‐VP groups. (F) Enrichment analysis of DEGs between M‐PP and M‐VP. (G) Heatmap of DEGs between M‐PP and M‐VP. (H) M1/M2 marker expression and cytokine secretion profiles in M‐VN, M‐VP, M‐PN, and M‐PP. (I) M1/M2 marker expression and cytokine secretion profiles in PIR‐B^−^ and PIR‐B^+^ uterine macrophages at GD6.5 during first (B^−^: PIR‐B^−^ macrophages, B^+^: PIR‐B^+^ macrophages) and second (B‐B^−^: PIR‐B^−^ macrophages, B‐B^+^: PIR‐B^+^ macrophages) pregnancies. Data in H, I are presented as mean ± SEM. Statistical differences were determined by one‐way ANOVA in H, I; ^*^ *p* < 0.05, ^**^ *p* < 0.01, ^***^ *p* < 0.001, ^****^ *p* < 0.0001.

Parallel analysis in mice demonstrated that PIR‐B^+^ uterine macrophages shared conserved transcriptional and functional signatures with human LILRB3^+^ DMs. RNA sequencing of PIR‐B^−^ and PIR‐B^+^ macrophages from virgin (M‐VN, M‐VP) and postpartum (M‐PN, M‐PP) mice revealed enrichment of M2‐polarization–related genes (Figure 5A–D). *Pirb* deficiency reduced the expression of M2‐associated transcripts (Figure 5B), whereas PIR‐B^+^ macrophages from postpartum (M‐PP) upregulated Art1, Spock1, Mmp28, Ror1, Bnc1, Notch3, and Foxh1, all implicated in macrophage polarization and pregnancy maintenance (Figures 5E–G). Metabolic genes such as Aldh1l2, Cox8b, Atp2b2, and Cidec were also elevated, indicating coordinated metabolic reprogramming (Figure 5F,G).

Phenotypically, PIR‐B^+^ macrophages displayed reduced CD86 and TNF‐α and increased CD206, TGF‐β, and IL‐10 compared with PIR‐B^−^ subsets (Figure 5H,I). These features were amplified in macrophages from second pregnancies, consistent with a trained tolerance phenotype.

To test whether this enhancement was paternal antigen–specific, we analyzed macrophages from syngeneic and allogeneic mating. The PIR‐B^+^ subset consistently exhibited a more tolerogenic profile—lower CD86, higher CD206, TGF‐β, and IL‐10—than PIR‐B^−^ macrophages (Figure S3A). Upon re‐exposure to the same paternal antigen, tolerogenic markers were further elevated: CD206 increased in B‐B^+^ mice and IL‐10 in C‐C^+^ mice, whereas this enhancement was absent in syngeneic and antigen‐switch mating (Figure S3B). *Pirb^−^/^−^* females showed no such differences between first and second pregnancies (Figure S3C), confirming that PIR‐B is indispensable for antigen‐driven macrophage reprogramming.

Together, these findings demonstrate that LILRB3/PIR‐B marks a conserved pregnancy‐induced macrophage memory program that promotes immune tolerance and tissue adaptation in subsequent pregnancies through antigen‐dependent, macrophage‐intrinsic mechanisms.

### 2.4 PIR‐B Mediates Metabolic Reprogramming in Macrophages to Sustain Pregnancy‐Induced Immune Memory

**FIGURE 6:**
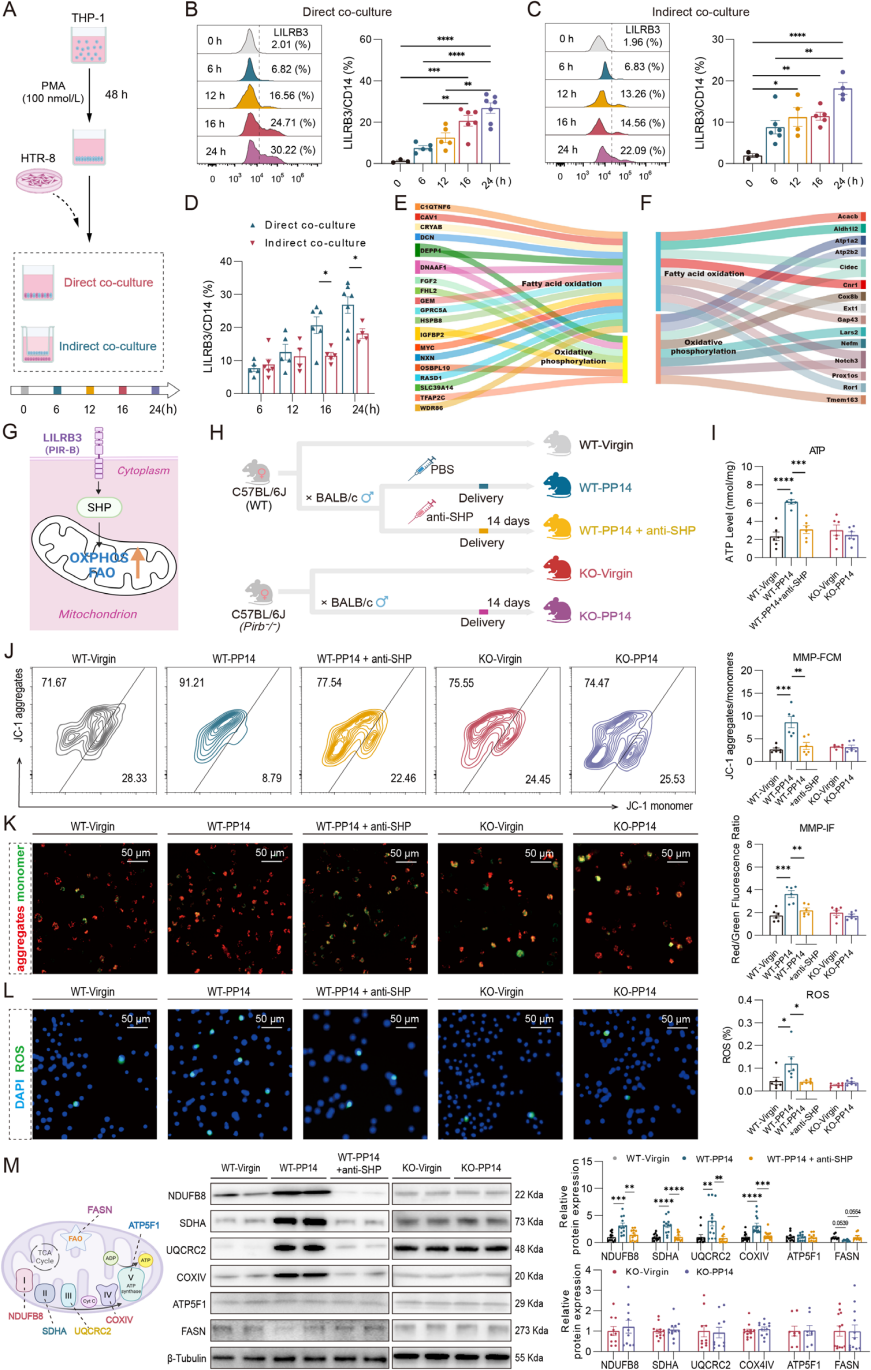
Mechanism of pregnancy immune memory formation in macrophages at the maternal‐fetal interface. (A) Schematic of HTR‐8/SVneo and THP‐1 coculture system. (B and C) Time‐dependent LILRB3 expression in THP‐1 after (B) direct or (C) indirect coculture with HTR‐8/SVneo. (D) LILRB3 expression in THP‐1 under direct vs indirect coculture. (E) Transcriptome analysis: human MP vs PP. (F) Transcriptome analysis: mouse M‐PP vs M‐VP. (G) Proposed pathway for pregnancy immune memory generation in macrophages. (H) Mouse model for mechanistic validation. (Abbreviations: WT‐Virgin, wild‐type virgin mice; WT‐ PP14, wild‐type mice at PP14; WT‐PP14 + anti‐SHP, wild‐type mice at PP14 with SHP blockade during pregnancy; KO‐Virgin, *Pirb^−/−^* virgin mice; KO‐PP14, *Pirb* ^−^/^−^ mice at PP14.) (I) ATP levels in BMDMs. (J and K) FCM (J) and IF (K) detection of mitochondrial membrane potential (MMP) in BMDMs (red: aggregates, green: monomer). Scale bar, 50 µm. (L) IF detection of reactive oxygen species (ROS) in BMDMs (green: ROS, blue: DAPI). Scale bar, 50 µm. (M) Expression of metabolism‐related proteins (Complex I–V: NDUFB8, SDHA, UQCRC2, COX4I1, ATP5F; fatty acid synthesis: FASN). Data in B‐D, I‐M are presented as mean ± SEM. Statistical differences were determined by unpaired two‐tailed t‐test in D, I‐M and by one‐way ANOVA in B, C, I‐M; ^*^ *p* < 0.05, ^**^ *p* < 0.01, ^***^ *p* < 0.001, ^****^ *p* < 0.0001.

To investigate whether trophoblasts drive macrophage reprogramming during pregnancy, we cocultured HTR‐8/SVneo trophoblasts with THP‐1–derived macrophages (Figure 6A). LILRB3 expression in THP‐1 cells increased in a time‐dependent manner following coculture, with greater induction under direct cell‐contact conditions (Figure 6B–D), indicating that trophoblast–macrophage interactions promote sustained activation of the LILRB3 axis.

To identify conserved intracellular mechanisms underlying macrophage memory, we conducted integrated transcriptional profiling of human (MP vs. PP) and mouse (M‐PP vs. M‐VP) macrophages. Both datasets revealed coordinated upregulation of OXPHOS and fatty acid oxidation (FAO) genes (Figure 6E,F), suggesting a shared metabolic rewiring across species. Because SHP functions as a canonical downstream effector of LILRB/PIR‐B signaling [34], we used mouse models to test whether the PIR‐B–SHP axis drives this metabolic adaptation (Figure 6G,H).

Bone marrow–derived macrophages (BMDMs) from postpartum wild‐type (WT‐PP14) mice exhibited a pronounced shift toward OXPHOS/FAO metabolism, characterized by upregulated mitochondrial genes, reduced lipogenesis (Fasn), elevated ATP production, enhanced mitochondrial membrane potential (MMP), and increased reactive oxygen species (ROS), accompanied by reduced glucose utilization and stable lactate output compared with virgin controls (WT‐virgin) (Figure 6I–L and Figure S4A,D–G).

In contrast, *Pirb^−^/^−^* postpartum (KO‐PP14) macrophages failed to undergo this metabolic reprogramming, displaying increased glycolytic gene expression and lack of OXPHOS/FAO induction relative to KO‐virgin BMDMs (Figure 6I–L and Figure S4B,D–G). Moreover, pharmacologic inhibition of SHP during pregnancy (WT‐PP14 + anti‐SHP) abrogated the OXPHOS/FAO gene signature, resulting in reduced ATP and MMP, lower ROS, and upregulated Fasn, closely resembling the metabolic profile of virgin macrophages (Figure 6I–L and Figure S4C,D–G).

Western blot analysis confirmed elevated expression of mitochondrial complexes I–IV (OXPHOS machinery) and suppression of FASN in WT‐PP14 BMDMs relative to WT‐virgin and WT‐PP14 + anti‐SHP groups (Figure 6M). These mitochondrial and lipid metabolic changes were absent in *Pirb^−^/^−^* macrophages, regardless of pregnancy status (Figure 6M).

Collectively, these findings demonstrate that PIR‐B–SHP signaling orchestrates a metabolic transition from glycolysis to OXPHOS and FAO, establishing a bioenergetic foundation for macrophage immune memory during pregnancy. This metabolic rewiring represents a critical mechanism by which macrophages acquire and sustain pregnancy‐induced tolerance across gestations.

### 2.5 Adoptive Transfer of PIR‐B^+^ Uterine Macrophages Improves Pregnancy Outcomes in Abortion‐Prone Mice

**FIGURE 7:**
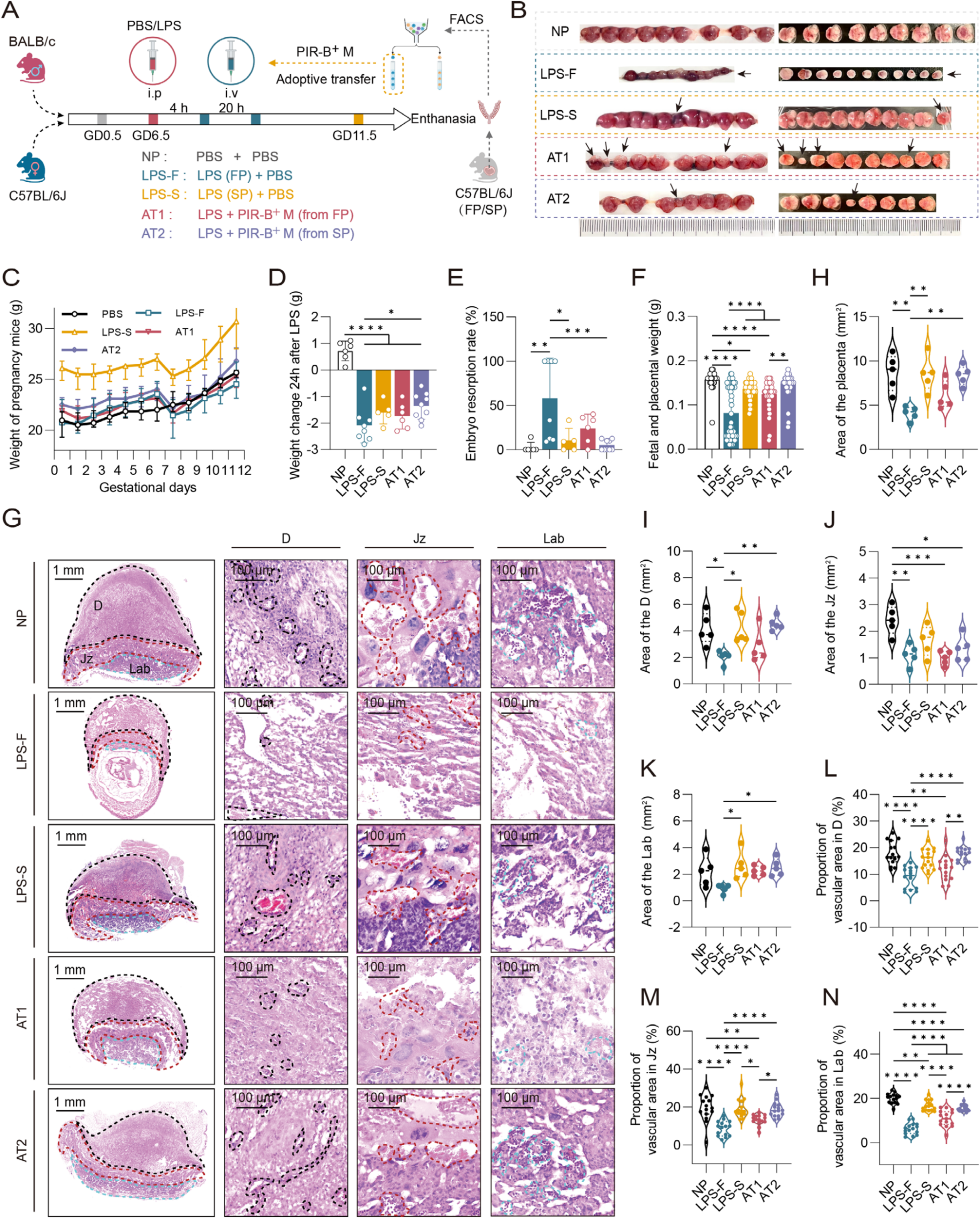
Adoptive transfer of PIR‐B^+^ uterine macrophages rescue pregnancy in abortion‐prone mice. (A) Experimental design for adoptive transfer in abortion‐prone model. (B) Representative uterine images. (C) Maternal body weight change during pregnancy. (D) Maternal weight changes 24 h after PBS/LPS injection. (E) Embryo resorption rate. (F) Fetal‐placental weight. (G) H&E staining of placental sections. Scale bars, 1 mm (overview), 100 µm (detail). (H) Total placental area. (I–K) Area of D (I), Jz (J), and Lab (K). (L–N) Vascular area percentage in D (L), Jz (M), and Lab (N). Data in C‐F, H‐N are presented as mean ± SEM. Statistical differences were determined by one‐way ANOVA in D‐F, H‐N; ^*^ *p* < 0.05, ^**^ *p* < 0.01, ^***^ *p* < 0.001, ^****^ *p* < 0.0001.

To assess the functional role of PIR‐B^+^ uterine macrophages in maintaining pregnancy tolerance, we employed an LPS‐induced abortion‐prone model during either the first (LPS‐F) or second (LPS‐S) pregnancy, alongside a normal pregnancy (NP) control (Figure 7A). PIR‐B^+^ uterine macrophages isolated from first (AT1) or second (AT2) pregnancies were adoptively transferred into LPS‐challenged primigravid mice at 4 and 24 h after LPS exposure (Figure 7A).

LPS challenge triggered maternal weight loss within 24 h, which was significantly attenuated in mice receiving AT2 macrophages (Figure 7B–D). Consistent with this, the embryo resorption rate was markedly increased in the LPS‐F group compared with NP controls, whereas LPS‐S and AT2 recipients displayed substantially reduced resorption (Figure 7E). Fetal–placental weights were significantly higher in the LPS‐S, AT1, and AT2 groups than in LPS‐F, with the AT2 group exhibiting the greatest recovery (Figure 7F).

Histological analysis of placentas at GD11.5 revealed severe structural disruption in the LPS‐F group, including loss of total placental area, decidual (D), junctional zone (Jz), and labyrinth (Lab) compartments, accompanied by impaired vascularization (Figure 7G–N). In contrast, both the LPS‐S and AT2 transfer groups exhibited attenuated placental injury, with restored architecture, expanded placental compartments, and enhanced vascular regeneration (Figure 7G–N). Notably, AT2‐derived macrophage transfer resulted in significantly greater vascular recovery than AT1 transfer across all placental zones (Figure 7L–N).

Together, these findings demonstrate that prior pregnancy confers protective immunity against inflammatory pregnancy loss and that PIR‐B^+^ uterine macrophages from second pregnancies actively mediate placental repair and fetal survival. These results identify PIR‐B^+^ macrophages as effector cells of pregnancy‐induced immune memory, capable of transferring tolerance and resilience to inflammation‐associated pregnancy complications.

## 3 Discussion

We report for the first time that pregnancy imprints a durable form of tissue‐resident immune memory within the uterus, mediated by a specialized subset of macrophages that expands across gestations and enhances fetal fitness. We identify LILRB3^+^ (human) and PIR‐B^+^ (mouse) uterine macrophages as a conserved memory‐competent population that emerges only after pregnancy, increases with repeated exposure to the same paternal antigens, and persists postpartum in a manner analogous to trained immunity. This macrophage adaptation is both tissue‐restricted and pregnancy‐dependent, linking reproductive history to improved placental development, fetal growth, and resilience to inflammatory stress in subsequent pregnancies.

**FIGURE 8:**
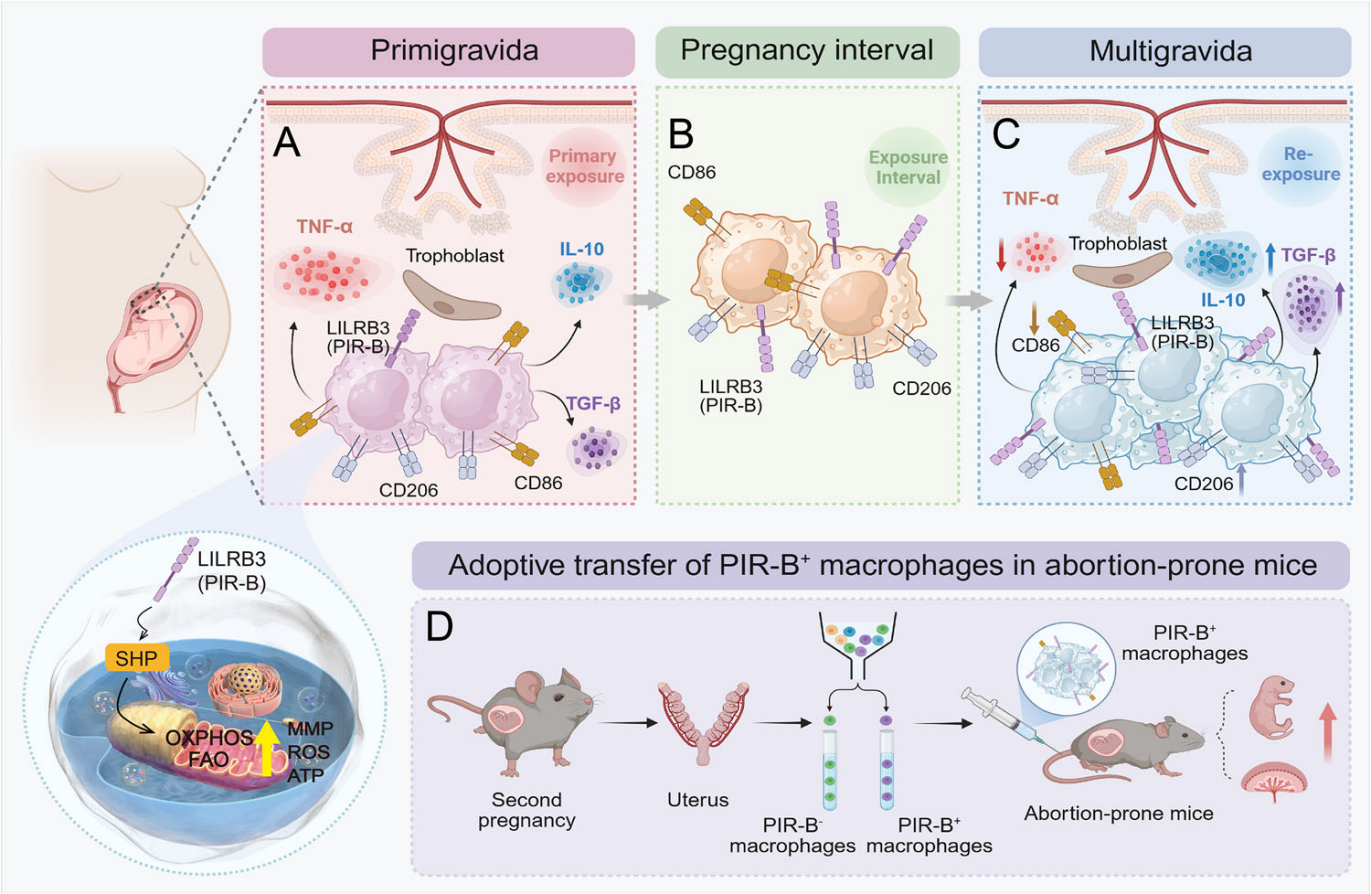
Pregnancy induces a unique population of memory LILRB3/PIR‐B^+^ uterine macrophages that are beneficial for subsequent pregnancies. (A) During the first pregnancy, trophoblasts induce LILRB3/PIR‐B to recruit SHP, which enhances mitochondrial OXPHOS and FAO, thereby driving the establishment of immunological memory in LILRB3/PIR‐B^+^ macrophages. (B) LILRB3/PIR‐B^+^ macrophages with immunological memory are maintained in the uterus after delivery. (C) Upon re‐exposure to pregnancy stimuli in a subsequent gestation, the LILRB3/PIR‐B^+^ macrophage population rapidly expands and shifts toward a more tolerogenic phenotype, characterized by increased secretion of IL‐10 and TGF‐β, which contributes to improved outcomes in the subsequent pregnancy. (D) Adoptive transfer of PIR‐B^+^ uterine macrophages from the second pregnancy rescued adverse pregnancy outcomes in abortion‐prone mice. Created with BioRender.

Macrophages, as the second most abundant immune cell population at the maternal‐fetal interface, play pivotal roles in pregnancy, including pathogen defense, maternal‐fetal immune tolerance maintenance, trophoblast invasion promotion, and spiral artery remodeling [35]. The concept of “trained immunity,” first introduced in 2011, has expanded our understanding of macrophage functionality, demonstrating that macrophages, similar to adaptive immune cells, can develop immunological memory via metabolic and epigenetic reprogramming [21]. However, whether macrophages at maternal‐fetal interface acquire pregnancy‐specific immune memory remains unclear. In this study, we addressed this gap by identifying a unique pregnancy‐induced subset of LILRB3/PIR‐B^+^ macrophages in both humans and mice, which exhibited paternal specific immune memory. Further in vivo investigations suggested that the formation of this memory depends on the SHP–OXPHOS/FAO axis. Adoptive transfer of PIR‐B^+^ macrophages into abortion‐prone mouse models significantly improved pregnancy outcomes, highlighting their therapeutic potential (Figure 8). Our findings thus reveal a novel mechanism of innate immune memory in pregnancy.

LILRB is a group of inhibitory receptors widely expressed in a variety of human immune cells, including monocytes, macrophages, NK cells, T cells, and B cells. It includes 5 subtypes, namely, LILRB1‐5, which can inhibit immune cell activation through immunoreceptor tyrosine‐based inhibitory motif (ITIM) signaling [36]. Recent studies have revealed that LILRB is also enriched in decidual immune cells and stromal cells at the maternal–fetal interface, where they contribute to immune tolerance and vascular development [31]. LILRB1, for example, engages trophoblast HLA‐G to suppress dNK‐mediated cytotoxicity and promote secretion of growth factors [37, 38], while LILRB2 has been implicated in vascular remodeling of placenta [39]. In our analysis of DMs across gestation and gravidity, LILRB1 expression remained low and unchanged, and LILRB2 showed only a transient increase in multigravid first‐trimester samples. In contrast, LILRB3^+^ macrophages expanded markedly in multigravid women and continued to increase with advancing gestation, identifying LILRB3 as the dominant gravidity‐associated receptor in human DMs. This pattern parallels the expansion of pregnancy‐trained NKG2C^+^LILRB1^+^ dNK cells reported in multigravid women [32], suggesting a coordinated innate memory network at the maternal–fetal interface. Transcriptomic analyses further revealed that, compared with LILRB3^+^ DMs from first pregnancies, those from subsequent pregnancies exhibited upregulation of genes associated with M2 macrophage polarization (e.g., IGFBP2, THBS2, SPON2), tissue‐resident macrophage homeostasis (e.g., CD24, LUM, CTGF) as well as key proliferation‐related genes (e.g., H19, GAS1, BCAR1, IGFBP6). These findings suggest enhanced proliferative capacity, immune tolerance and local maintenance of LILRB3^+^ DMs during subsequent pregnancies.

In mice, PIR‐B functions as the closest homolog of human LILRB1–LILRB3 and similarly mediates inhibitory signaling via ITIM motif‐dependent recruitment of SHP [31]. PIR‐B^+^ uterine macrophages comprised approximately 5% of total macrophages in the nonpregnant state, gradually increased during pregnancy, and remained significantly elevated after the first pregnancy. This sustained post pregnancy enrichment supports the notion of stable in situ retention rather than transient recruitment alone. This kinetics parallels epidemiologic observations that the protective effects of prior pregnancy wane as inter‐pregnancy intervals lengthen [33, 40]. Notably, PIR‐B^+^ uterine macrophages expanded more rapidly and reached higher levels during second pregnancies, whereas splenic macrophages showed no comparable change, indicating a tissue‐restricted memory program.

Clinical epidemiological evidence has established that the protective effects of a prior normal pregnancy display strict partner specificity. Parous women who conceive with a new partner have a risk of PE (3.9%) comparable to that of nulliparous women, whereas the risk is significantly reduced to 1.9% in parous women who remain with the same partner [41]. Consistent with these clinical observations, Rowe and colleagues reported in a murine model that fetal antigen‐specific FOXP3^+^ Tregs persisted postpartum and only underwent rapid clonal expansion to confer protection against fetal loss upon re‐exposure to identical paternal antigens [18]. Similarly, Thiele et al. [17] confirmed that the functional benefits of paternal antigen‐induced CD4^+^ memory Tregs—including enhanced fetal growth and reduced miscarriage rates—were abrogated entirely following paternal replacement. These findings raise a key question: whether pregnancy‐induced immune memory in macrophages is also characterized by antigen‐specific recall responses, a hypothesis strongly supported by work from Dai et al. [30]. Their study definitively demonstrated that murine PIR‐B mediates antigen‐specific tolerogenic memory to MHC class I antigens in myeloid cells [30]. Building on this foundational evidence, we designed our experiments using male mice of distinct strains, which express divergent H‐2 MHC haplotypes and thus ensure the uniqueness of paternal antigen repertoires.

Because PIR‐B has been linked to antigen‐specific macrophage memory and pregnancy outcomes are known to depend on paternal antigens, we tested whether this memory was sire‐specific. Syngeneic second pregnancies failed to induce PIR‐B^+^ macrophage expansion, whereas repeated exposure to the same allogeneic sire (B→B or C→C) drove robust PIR‐B accumulation and improved fetal–placental growth. Antigen‐switch (B→C) and interrupted‐exposure (B→C→B) mating abolished both macrophage expansion and fetal benefits, demonstrating strict paternal antigen specificity. *Pirb*‐deficient females exhibited impaired fetal development that worsened in second pregnancies, confirming PIR‐B's functional requirement. Future macrophage‐specific *Pirb* deletion models will be necessary to definitively attribute this phenotype to uterine macrophages. In addition, to further characterize the fetal‐antigen specificity of PIR‐B^+^ uterus macrophage memory, the surrogate antigen strategy of Rowe et al. [18] could be adopted in future study, which utilizes MHC class II tetramer‐based enrichment to identify the surrogate antigen.

Mechanisms underlying pregnancy‐induced immune memory remain unclear. Carrying paternal antigens, trophoblasts are the sole fetal‐derived cells that establish direct contact with maternal immune cells at the maternal–fetal interface [42]. We therefore performed trophoblast‐macrophage coculture, and found that the human trophoblast cell line HTR‐8/SVneo induced LILRB3 expression in THP‐1 macrophages. This suggests trophoblasts may prime macrophage memory through antigen‐driven activation, a finding that requires further validation. Metabolic reprogramming is one of the core mechanisms of trained immunity across innate immune cells, providing the energetic and biosynthetic foundation for immune function [43]. In macrophages, metabolic state tightly dictates polarization: M1‐like inflammatory macrophages favor glycolysis and succinate accumulation, whereas M2‐like regulatory macrophages depend on fatty‐acid oxidation (FAO) and OXPHOS to sustain reparative functions. Our RNA‐seq analysis identified upregulated OXPHOS and FAO‐related genes in human and mouse LILRB3^+^/PIR‐B^+^ macrophages following pregnancy. In mice, we further found that the PIR‐B–SHP axis serves as a key mediator of these metabolic changes. This mirrors findings in IL‐4/IL‐13 research, which show that activated macrophages form protective innate memory against M. tuberculosis reliant on sustained high OXPHOS [44]. High OXPHOS/FAO activity, whose derived metabolites such as α‐ketoglutarate and succinate promote M2 macrophage polarization and tolerance via metabolic reprogramming, is well established to correlate with M2‐polarized, tolerogenic macrophage phenotypes [45, 46]. These findings mechanistically explain reinforced uterine macrophage‐mediated immune tolerance post‐pregnancy. We propose that the OXPHOS/FAO shift provides a persistent metabolic substrate that sustains long‐term tolerogenic function and rapid recall.

The translational potential based on pregnancy‐induced macrophage memory is evidenced by our adoptive transfer studies in mice, wherein PIR‐B^+^ uterine macrophages from secondary pregnancies conferred markedly more robust protection against LPS‐induced fetal loss compared to their first‐pregnancy counterparts. This enhanced protective effect is likely attributable to improved placental development. In LPS‐challenged mice, adoptive transfer of PIR‐B^+^ uterine macrophages isolated from secondary pregnant donors robustly restored vascular area across all three placental compartments, with the most striking restorative effect evident in the labyrinth layer—the primary anatomical site mediating bidirectional maternofetal material exchange. Notably, the murine labyrinth layer forms a dense vascular plexus that underpins efficient materno‐fetal transport, while the junctional zone mediates nutrient exchange and synthesizes pregnancy‐associated hormones. Collectively, these findings establish PIR‐B^+^ uterine macrophages as active effectors—rather than passive biomarkers—of pregnancy tolerance, thereby highlighting their considerable therapeutic potential for the management of inflammatory and immune‐mediated pregnancy complications (e.g., recurrent pregnancy loss (RPL), PE). Conversely, whether insufficient LILRB3^+^ DMs contribute to human miscarriage, and their translational potential for clinical application, remain to be fully elucidated in well‐powered human cohort studies.

Although this work defines a conserved macrophage memory program in humans and mice, several key questions remain. Because our studies relied on global *Pirb* knockout mice, macrophage‐specific conditional deletion will be essential to confirm the cell‐autonomous role of PIR‐B in maternal immune regulation. In addition, the upstream signals that initiate and sustain LILRB3/PIR‐B activation remain unresolved. We propose that trophoblast‐derived ligands may serve as the imprinting signal, and identification of the human LILRB3 ligand will be critical for translating these findings into targeted interventions.

Together, our findings reveal that pregnancy imprints a durable, pregnancy‐specific form of innate immune memory in uterine macrophages, marked by the emergence of LILRB3^+^/PIR‐B^+^ cells that expand across gestations, acquire a tolerogenic and metabolically rewired phenotype, and actively protect against inflammatory pregnancy loss. This work reframes the maternal–fetal interface as a site where prior reproductive experience reshapes innate immunity to optimize subsequent pregnancies, establishing macrophage memory as a previously unrecognized determinant of placental development and fetal fitness. By identifying a conserved, targetable pathway for inducing or restoring this memory, our study opens new therapeutic avenues for RPL, PE, and other immune‐mediated gestational disorders, while laying the foundation for future efforts to manipulate trained immunity in reproductive health.

## 4 Materials and Methods

### 4.1 Human Decidual Sample Collection

Human samples were collected from participants recruited at the Hubei Maternal and Child Health Hospital and Liyuan Hospital between June 2021 and June 2024. Written informed consent was obtained from all participants prior to sample collection. All experiments involving human were conducted according to the ethical policies and procedures approved by Clinical Trial Ethics Committee of Huazhong University of Science and Technology, Wuhan, China (Approval no. CTEC‐S154).

First‐trimester decidual samples were obtained from healthy women undergoing voluntary termination of normal pregnancies (4–12 weeks). Maternal age averaged 26 years (range: 17–37 years) in primigravid and 29 years (range: 19–42 years) in multigravid women. Detailed cohort characteristics are provided in Table S1. Detailed information on first‐trimester decidual samples used for phenotype and cytokine detection is provided in Table S4.

Third‐trimester decidual samples were obtained from healthy women delivering at term (37–41 weeks) of uncomplicated pregnancies. Exclusion criteria included those applied to the first‐trimester cohort, as well as pregnancy complications and placental abnormalities. Maternal age averaged 22 years (range: 20–24 years) in primigravid and 30 years (range: 26–39 years) in multigravid women. Detailed cohort characteristics are provided in Table S2.

### 4.2 Mice

C57BL/6J (H‐2b), BALB/c (H‐2d), and C3H/HeJ (C3H, H‐2k) mice (6–8 weeks old) were purchased from Shulaibao Biotechnology Co., Ltd. (Wuhan, China). *Pirb* ^−^/^−^ mice with a C57BL/6J background were obtained from Cyagen Biosciences (Guangzhou, China). Genotypes of *Pirb* ^−^/^−^ mice were verified by PCR using specific primers (sequences provided in Table S7) prior to experimentation. All mice were maintained under specific pathogen‐free conditions in the Animal Research Center of Tongji Medical College, with a controlled environment (22 ± 2°C, 50 ± 5% humidity, 12 h light/dark cycle). Animal experiments were approved by the Institutional Animal Care and Use Committee of Huazhong University of Science and Technology and complied with ethical regulations. All experiments involving animals were conducted according to the ethical policies and procedures approved by the Institutional Animal Care and Use Committee of Tongji Medical College, Huazhong University of Science and Technology, Wuhan, China (Approval no. IACUC‐5141).

### 4.3 Cell Lines and Culture

The human trophoblast cell line HTR‐8/SVneo and the monocytic leukemia cell line THP‐1 were obtained from the China Center for Type Culture Collection (CCTCC, Wuhan, China). Both cell lines were cultured in RPMI 1640 medium (Gibco, USA) supplemented with 10% fetal bovine serum (Gibco, USA) and 1% penicillin‐ streptomycin at 37°C in a humidified atmosphere of 5% CO_2_. The culture medium was refreshed every 2–3 days. HTR‐8/SVneo cells were passaged using 0.25% trypsin‐EDTA (Gibco, USA) when cell confluence reached 80–90%. THP‐1 cells were maintained in suspension and subcultured by dilution. Standard aseptic techniques were followed throughout all cell culture procedures.

### 4.4 Isolation of Human Decidual Macrophages (DMs)

Decidual tissues were rinsed with PBS and minced into 1–3 mm^3^ fragments on ice. The tissue fragments were digested for 1 h at 37°C in a solution containing Collagenase IV (1 mg/mL; BioFroxx, Germany), Hyaluronidase (1 mg/mL; Sigma–Aldrich, USA), and DNase I (150 µg/mL; Sigma–Aldrich, USA). The digest mixture was filtered through an 80‐µm nylon mesh, and the enzymatic reaction was quenched by adding PBS containing 2% FBS. This mixture was centrifuged at 800×g for 10 min at 4°C, and the cell pellet was washed twice with PBS. The cells were then treated with 2 mL of red blood cell lysis buffer (Biosharp, China) and incubated for 5–10 min at 4°C. The lysis was stopped with PBS, and the cells were washed twice. Cell pellet was then resuspended in 25% Percoll solution (Biosharp, China), carefully layered onto a 50% Percoll solution, and topped with 2 mL of PBS. This discontinuous gradient was centrifuged at 800×g for 20 min at 4°C without brake. Mononuclear cells at the interface between the 25% and 50% Percoll layers were collected, diluted with an equal volume of PBS, washed, and passed through a 300‐mesh cell strainer. After a final centrifugation at 500×g for 5 min, the cell pellet was resuspended in separation buffer for flow cytometry (FCM) for subsequent staining.

### 4.5 Multicolor FCM

Single‐cell suspensions were stained with fluorochrome‐conjugated anti‐human or anti‐mouse antibodies (detailed in Table S5). Human macrophages were defined as CD14^+^ cells, whereas mouse macrophages were identified as CD45^+^F4/80^+^. For intracellular cytokine staining (TNF‐α, TGF‐β, IL‐10, and IFN‐γ), cells were stimulated with Cell Activation Cocktail (BioLegend, USA) for 4.5 h. Stimulated cells were then fixed and permeabilized using a commercial kit (BD Biosciences, USA). Stained samples were acquired using a NovoCyte flow cytometer (Agilent). Data were analyzed using NovoCyte 2.0 software.

### 4.6 Tissue Paraffin Embedding and Sectioning

Human decidual tissues and mouse placentas were fixed in 4% paraformaldehyde (Biosharp, China) for 24 h at room temperature. Embedding cassettes containing fixed tissues were removed from the fixative and rinsed under running tap water for 15 min. The tissues were then dehydrated through a graded ethanol series (50%, 70%, 80%, 95%, 100%, 100%; 1 h each), cleared in xylene, infiltrated with paraffin, and embedded. The resulting paraffin blocks were hardened at ‐20°C for 4 h prior to sectioning into 3–5 µm slices using a microtome. Sections were floated on a 42°C water bath to spread, transferred onto adhesion slides, dried overnight at 37°C, and stored at room temperature for subsequent use.

### 4.7 Haematoxylin–Eosin (HE) Staining

Mouse placental sections were baked at 60°C for 2 h. Baked sections were dewaxed in xylene through four 10‐min incubations. Dewaxed sections were rehydrated in a descending ethanol series (100%, 90%, 80%, and 70%; 10 min each). Then, the sections were stained with hematoxylin (Servicebio, China) for nuclear staining for 3 min, followed by rinsing with running water to remove excess hematoxylin solution. After that, the sections were differentiated with 1% hydrochloric acid‐ethanol for 5 s and rinsed with running water again. They were then blued in weak ammonia water for 5 s, followed by another rinse with running water. Next, the sections were stained with eosin solution for 30 s. Finally, the sections were dehydrated through a graded ethanol series, cleared in xylene, mounted with neutral balsam, and observed and photographed.

### 4.8 Immunohistochemistry (IHC)

Human decidual tissue sections were dewaxed, rehydrated, and subjected to heat‐induced antigen retrieval in preheated Tris‐EDTA buffer (pH 9.0; Beyotime, China) at high power for 15 min, followed by natural cooling to room temperature. After three 5‐min washes with PBS, the sections were blocked with goat serum (Boster, China) at room temperature for 1 h. The sections were then incubated with diluted LILRB3 primary antibody (Abcam, UK) in a humidified chamber at 4°C overnight. After rewarming at room temperature for 30 min and removal of the primary antibody, the sections were washed three times with PBST (5 min each). Subsequently, they were incubated with goat anti‐rat secondary antibody (Proteintech, USA) for 1 h at room temperature, followed by another three PBST washes. Color development was performed using DAB under microscopic monitoring and stopped by rinsing with running water. The sections were counterstained with hematoxylin, differentiated in 1% HCl‐ethanol, blued in weak ammonia water, dehydrated through an ethanol series, cleared in xylene, and mounted with neutral balsam for microscopic examination and imaging. Staining was analyzed using ImageJ.

### 4.9 Immunofluorescence (IF)

Human decidual tissue or mouse placental tissue sections were retrieved and sequentially subjected to dewaxing and rehydration. Afterward, antigen retrieval was performed in sodium citrate buffer (Servicebio, China) via microwave heating. Sections were blocked with normal goat/donkey serum (Boster, China), incubated overnight at 4°C with primary antibodies against LILRB3 (Invitrogen, USA), CD68 (Santa Cruz Biotechnology, USA), CD31 (Proteintech, USA), or alpha‐SMA (Abcam, UK), followed by incubation with fluorescent secondary antibodies and DAPI counterstaining. Images were acquired using a ZEISS LSM900 confocal microscope.

### 4.10 Cell Coculture

THP‐1 monocytes (1 × 10^6^ cells/mL) were induced to differentiate into macrophages by treatment with 100 nmol/L PMA (MCE, USA) for 48 h. The differentiated THP‐1 cells were then cocultured with HTR‐8/SVneo trophoblasts at a 1:1 ratio (1 × 10^5^ cells/mL) in transwell plates, which permitted either direct contact or indirect interaction between the two cell types. LILRB3 expression in THP‐1 cells was analyzed by FCM at the indicated time points (6, 12, 16, and 24 h).

### 4.11 Isolation and Culture of Bone Marrow‐Derived Macrophages (BMDMs)

Mice were euthanized by cervical dislocation, and their skin was disinfected with 75% ethanol. Hind limbs were exposed, and femurs aseptically excised. Isolated femurs were placed in a culture dish with sterile ice‐cold PBS, and adherent muscle tissue was carefully removed. Femurs were washed twice with sterile ice‐cold PBS, then transferred to a sterile mortar with 5 mL ice‐cold PBS, and gently ground to release bone marrow. Homogenate was filtered through a 70‐µm nylon cell strainer to remove bone fragments. Filtrate was centrifuged at 450×g for 10 min at 4°C. Supernatant was discarded, and cell pellet resuspended in 1 mL sterile red blood cell lysis buffer, incubated at room temperature for 30 s. Lysis was immediately stopped by adding ice‐cold complete medium, and cells re‐centrifuged at 450×g for 10 min at 4°C. Pellet was resuspended in complete medium with 20% L929 cell supernatant (as M‐CSF source) and passed through a 40‐µm nylon cell strainer. Filtered cell suspension was collected, counted, and seeded into 10‐cm dishes at 2 × 10^7^ cells/dish. Each dish was filled to 10 mL with complete medium containing 20% L929‐conditioned medium and cultured in a humidified 37°C, 5% CO_2_ incubator. On culture day 3, an additional aliquot of complete medium with 20% L929‐conditioned medium was added. Cell morphology was monitored microscopically; on day 5, adherent BMDMs were harvested for subsequent experiments.

### 4.12 Reactive Oxygen Species (ROS) Measurement

Cellular ROS levels were detected using the fluorescent probe DCFH‐DA (Beyotime, China). A working solution was prepared by diluting DCFH‐DA in PBS to a final concentration of 10 µmol/L. Prior to staining, the culture medium was aspirated from the wells containing induced BMDMs. The cells were gently washed with PBS, and then 1 mL of the DCFH‐DA working solution was added to each well. The plates were incubated at 37°C for 20 min in the dark. During incubation, the plates were gently swirled every 3–5 min to ensure uniform probe distribution. After incubation, the solution was removed, and the cells were washed three times with serum‐free medium to eliminate residual extracellular probe. ROS generation was qualitatively analyzed by laser scanning confocal microscopy.

### 4.13 Mitochondrial Membrane Potential (MMP) Measurement

MMP in BMDMs was assessed using the JC‐1 fluorescent probe and analyzed by both FCM and IF. A 1×working solution of JC‐1 was prepared in accordance with the manufacturer's protocol (Beyotime, China).

For IF, the culture medium was aspirated from 12‐well plates containing BMDMs, and the cells were gently washed with PBS. Each well was then treated with a mixture consisting of 0.5 mL fresh culture medium and 0.5 mL JC‐1 working solution. After gentle mixing, the plates were incubated at 37°C for 20 min in the dark, with gentle swirling every 3–5 min to ensure even dye distribution. Following incubation, the staining solution was removed, and the cells were washed twice with ice‐cold assay buffer. Finally, 1 mL of fresh culture medium was added, and the cells were immediately visualized using a laser scanning confocal microscope.

For FCM, BMDMs were collected and resuspended in 0.5 mL of culture medium. The cell suspension was mixed with 0.5 mL of JC‐1 working solution and incubated at 37°C for 20 min in the dark. During incubation, the tubes were gently inverted every 3–5 min. After incubation, the cells were centrifuged at 600×g for 5 min at 4°C. The pellet was washed twice with ice‐cold assay buffer, resuspended in 200 µL of the same buffer, and analyzed by FCM.

### 4.14 ATP Measurement

The culture medium was aspirated from 6‐well plates containing BMDMs, and the cells were gently washed with PBS. Then, 200 µL of lysis buffer was added to each well, and the cells were thoroughly lysed by repeated pipetting. The resulting cell lysates were collected and centrifuged at 12,000×g for 5 min at 4°C. The supernatant was subsequently collected for ATP quantification.

### 4.15 RNA Extraction and RT‐qPCR

Total RNA was extracted using TRIzol reagent according to the manufacturer's instructions. Genomic DNA was removed from 1 µg of total RNA using a gDNA Eraser reagent, and the RNA was subsequently reverse‐transcribed into cDNA in a 20 µL reaction system (Yeasen Biotech, China). Quantitative PCR was performed using 2 µL of cDNA with SYBR Green Master Mix (Yeasen Biotech, China) on a q225 Real‐Time PCR System (Kubo Technology, China). All reactions were run in duplicate, and gene expression levels were normalized to β‐actin. The primer sequences used were listed in Table S7.

### 4.16 Western Blotting

BMDMs were lysed using RIPA lysis buffer (Beyotime, China) supplemented with protease inhibitors and phosphatase inhibitors. After lysis, cell debris was removed, and the resulting supernatants were collected. Protein concentrations in the supernatants were determined using a BCA protein assay kit (Beyotime, China). Equal amounts of protein were mixed with SDS loading buffer, denatured, and then separated by SDS‐PAGE. Following electrophoresis, the separated proteins were transferred onto PVDF membranes. The membranes were blocked with 5% non‐fat milk to block non‐specific binding sites, and then incubated overnight at 4°C with primary antibodies. The next day, the membranes were washed to remove unbound primary antibodies, followed by incubation with HRP‐conjugated secondary antibodies. After additional washes, protein bands were visualized using an enhanced chemiluminescence detection system. The relative expression levels of target proteins were quantified by densitometric analysis of the bands using ImageJ software, with the intensity of each target band normalized to that of β‐tubulin. Anti‐human or anti‐mouse antibodies detailed in Table S6.

### 4.17 RNA Sequencing Data Analysis

Human LILRB3^−^ and LILRB3^+^ DMs (≥ 10^5^ cells) were isolated using a CytoFLEX SRT flow cytometer, lysed in TRIzol reagent, and immediately frozen on dry ice prior to shipment to Novogene (China) for sequencing. Detailed cohort characteristics are provided in Table S3. Mouse PIR‐B^−^ and PIR‐B^+^ uterine macrophages were processed identically. RNA extraction, library preparation, and subsequent bioinformatic analysis were performed by Novogene following standard protocols.

### 4.18 First and Second Pregnancy Models

Female C57BL/6J mice (6–8 weeks old) and male BALB/c mice (6–8 weeks old) were acclimatized for one week under controlled conditions. For mating, two females and one male were housed together daily from 20:00, and vaginal plugs were checked at 8:00 the next morning. The day of plug detection was designated as gestational day (GD) 0.5. Plug‐positive females were ear‐tagged, weighed, and housed individually.

The experiment included 4 main groups (12 subcategories total): virgin group (female mice without mating history); primary pregnancy group, divided into 4 subcategories by gestational days (GD6.5, GD11.5, GD16.5, GD18.5); postpartum group, subdivided into 3 subcategories by postpartum (PP) days (PP7, PP14, PP21); and secondary pregnancy group (mice remated with the same male BALB/c mouse between 14–21 days after their first parturition), split into 4 subcategories by secondary gestational days (GD6.5, GD11.5, GD16.5, GD18.5).

At scheduled time points, mice were euthanized by cervical dislocation. Uterine tissues (with placentas and fetuses) and spleen were collected, rinsed in ice‐cold PBS. Embryo resorptions were identified by reduced size or hemorrhagic spots. Fetal and placental weights were recorded. PIR‐B expression in uterine and splenic macrophages was analyzed by FCM.

### 4.19 Syngeneic and Allogeneic Pregnancy Models

All pregnancy models used female C57BL/6J mice. For syngeneic primary and secondary pregnancies, female C57BL/6J mice were paired with male C57BL/6J mice. Allogeneic pregnancies were established by mating female C57BL/6J mice with male BALB/c mice or male C3H/HeJ mice, respectively. Subgroups were named based on the number of pregnancies and the specific male mouse strain. For example, “C57‐C57” refers to both primary and secondary pregnancies where females were paired with C57BL/6J males. All aforementioned mice were euthanized at GD11.5 to evaluate pregnancy outcomes and the developmental status of fetuses and placentas. Spleen and uterus were collected. FCM was used to detect PIR‐B expression in macrophages. Additionally, one neonate was randomly selected from each group at GD18.5 for Alcian blue staining of the skeleton.

### 4.20 Mechanistic Validation Model

The experiment included five groups of female mice: the WT‐virgin group and KO‐virgin group consisted of unmated, untreated C57BL/6J wild‐type (WT) and C57BL/6J *Pirb*‐knockout (KO) female mice, respectively; the WT‐PP14 and KO‐PP14 groups were allogeneic pregnancy models generated by mating C57BL/6J WT and KO female mice with BALB/c male mice, respectively; and the WT‐PP14 + anti‐SHP group comprised C57BL/6J WT female mice mated with BALB/c male mice, which received daily intraperitoneal injections of 20 mg/kg SHP inhibitor (MCE, USA) starting on GD1.5 and continuing consistently until parturition to ensure sustained inhibitor exposure. The efficacy and safety of this inhibitor have been previously reported in the literature [47, 48]. All mice across the five groups were euthanized at GD11.5, followed by tissue processing: bone marrow cells were carefully isolated from the femurs of euthanized mice, induced to differentiate into BMDMs under standard in vitro culture conditions, and these induced BMDMs were subsequently used for a series of metabolism‐related detections.

### 4.21 Cell Adoptive Transfer Model

C57BL/6 female mice were mated with BALB/c male mice to establish a normal pregnancy control group. An abortion‐prone model of primary pregnancy was induced by intraperitoneal injection of LPS (2.5 µg/100 µL; Solarbio) on GD6.5. To establish a secondary pregnancy abortion‐prone model, C57BL/6 females that completed one normal pregnancy after mating with BALB/c males were remated with BALB/c males and administered LPS on GD6.5 of the subsequent pregnancy. To evaluate the therapeutic potential of uterine PIR‐B‐expressing macrophages, adoptive transfer was performed in primigravid mice subjected to LPS challenge on GD6.5. Specifically, PIR‐B‐expressing macrophages isolated from uteri of either primary or secondary pregnancy donors were transferred via tail vein injection at 4 and 24 h post‐LPS administration. All five experimental groups were euthanized on GD11.5 for assessment of pregnancy outcomes and fetal‐placental development.

### 4.22 Statistical Analysis

Data are presented as the mean ± SEM. All Statistical analyses were performed using GraphPad Prism 9.0 (GraphPad Software Inc., USA). A student's t‐test was used to determine the significance of differences between two groups. A one‐way analysis of variance (ANOVA) followed by Tukey's multiple comparisons test was used to compare multiple groups. Pearson's correlation analysis was used to analyze the linear relationship between two variables. *P* values < 0.05 were considered statistically significant.

## Author Contributions

Conceptualization: J.W. and A.H.L.; Methodology: J.W., N.L., X.H.F. and Y.T.P.; Investigation: J.W. and X.X.L.; Supervision: G.M. and A.H.L.; Writing – original draft: J.W., Q.P.L and Y.H.L.; Writing – review & editing: J.W., G.M., and N.L.

## Funding

This work was supported by the grant from the National Natural Science Foundation of China (No. 82220108008)

## Ethics Approvals

All experiments involving human were conducted according to the ethical policies and procedures approved by Clinical Trial Ethics Committee of Huazhong University of Science and Technology, Wuhan, China (Approval no. CTEC‐S154). All experiments involving animals were conducted according to the ethical policies and procedures approved by the Institutional Animal Care and Use Committee of Tongji Medical College, Huazhong University of Science and Technology, Wuhan, China (Approval no. IACUC‐5141).

## Conflicts of Interest

The authors declare no conflicts of interest.

## Supporting information




**Supporting File**: advs74889‐sup‐0001‐SuppMat.pdf.

## Data Availability

The data that support the findings of this study are available from the corresponding author upon reasonable request.
