# Supplementary material for: Trained Memory of Uterine Macrophages Improves Subsequent Pregnancy Outcomes
Source: Adv Sci (Weinh). 2026 Mar 28;13(31):e74889. doi: 10.1002/advs.74889 (PMC13587929; doi:10.1002/advs.74889)
Supplement: Supplementary file 1 [file ADVS-13-e74889-s001.pdf]

SUPPLEMENTAL INFORMATION

Trained memory of uterine macrophages improves subsequent pregnancy outcomes

Jing Wang[1], Ning Lu[1], Xin-Xiu Lin[1], Xu-Hui Fang[1], Yi-Hui Li[1], Qing-Peng Luo[1], Yu-Ting Peng[1], Gil Mor[1, 2], Ai-Hua Liao[1,]**

Affiliations:

[1]Institute of Reproductive Health, Center for Reproductive Medicine, Tongji Medical College, Huazhong University of Science and Technology, Wuhan, P.R. China

[2]C.S. Mott Center for Human Growth and Development, Wayne State University School of Medicine, Detroit, MI, USA

***Corresponding author:**

Ai-Hua Liao

E-mail address: aihua_liao@hust.edu.cn

***Co-corresponding author:**

Gil Mor

E-mail address: gmor@med.wayne.edu

This supplemental material includes:

Supplementary figure legends (Figures S1–S4)

Supplementary table 1-7

Supplementary figures

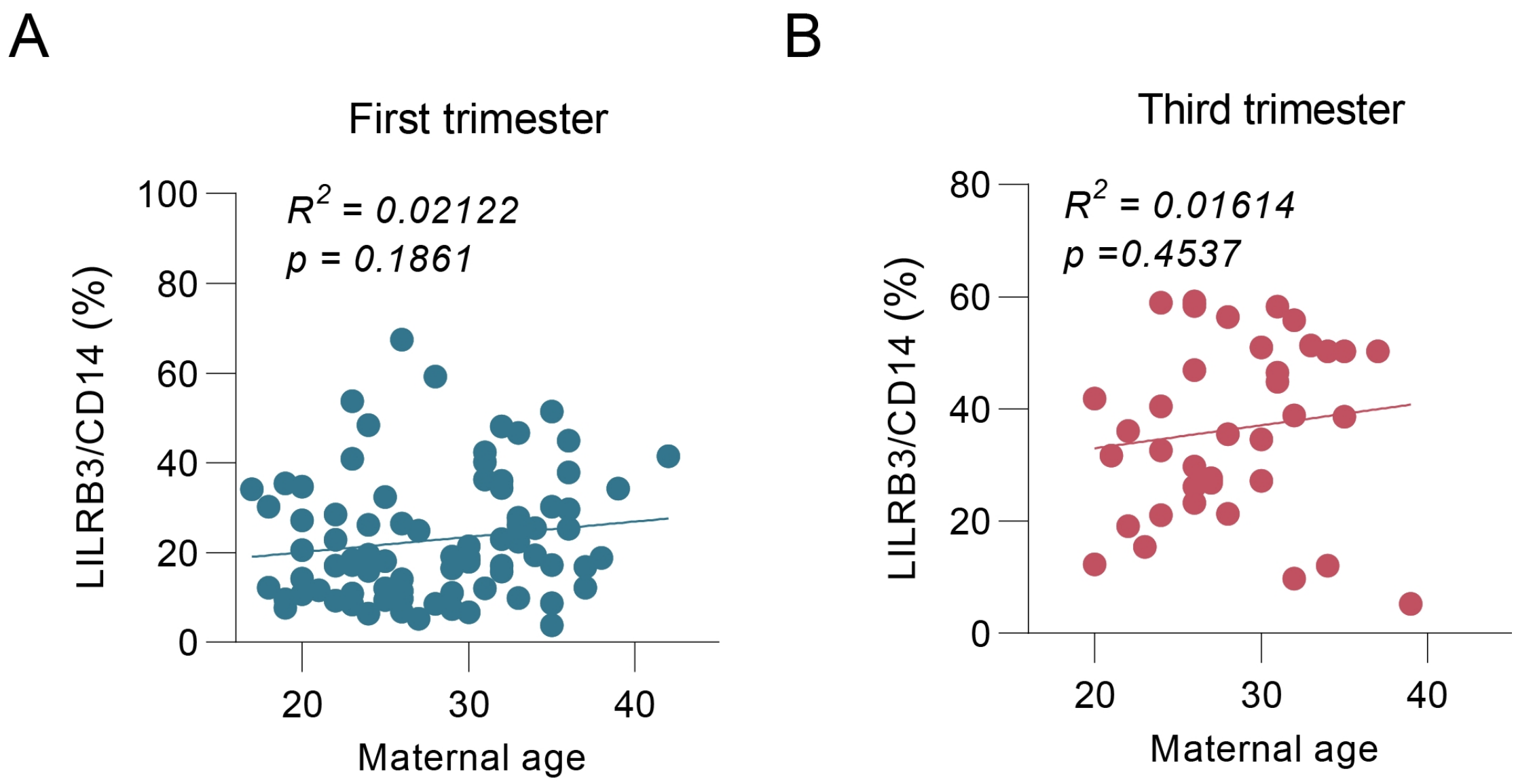

Supplemental Figure 1. Correlation between the proportion of LILRB3$^+$ decidual macrophages and maternal age. (A) First-trimester. (B) Third-trimester. Pearson correlation analysis was used in A and B.

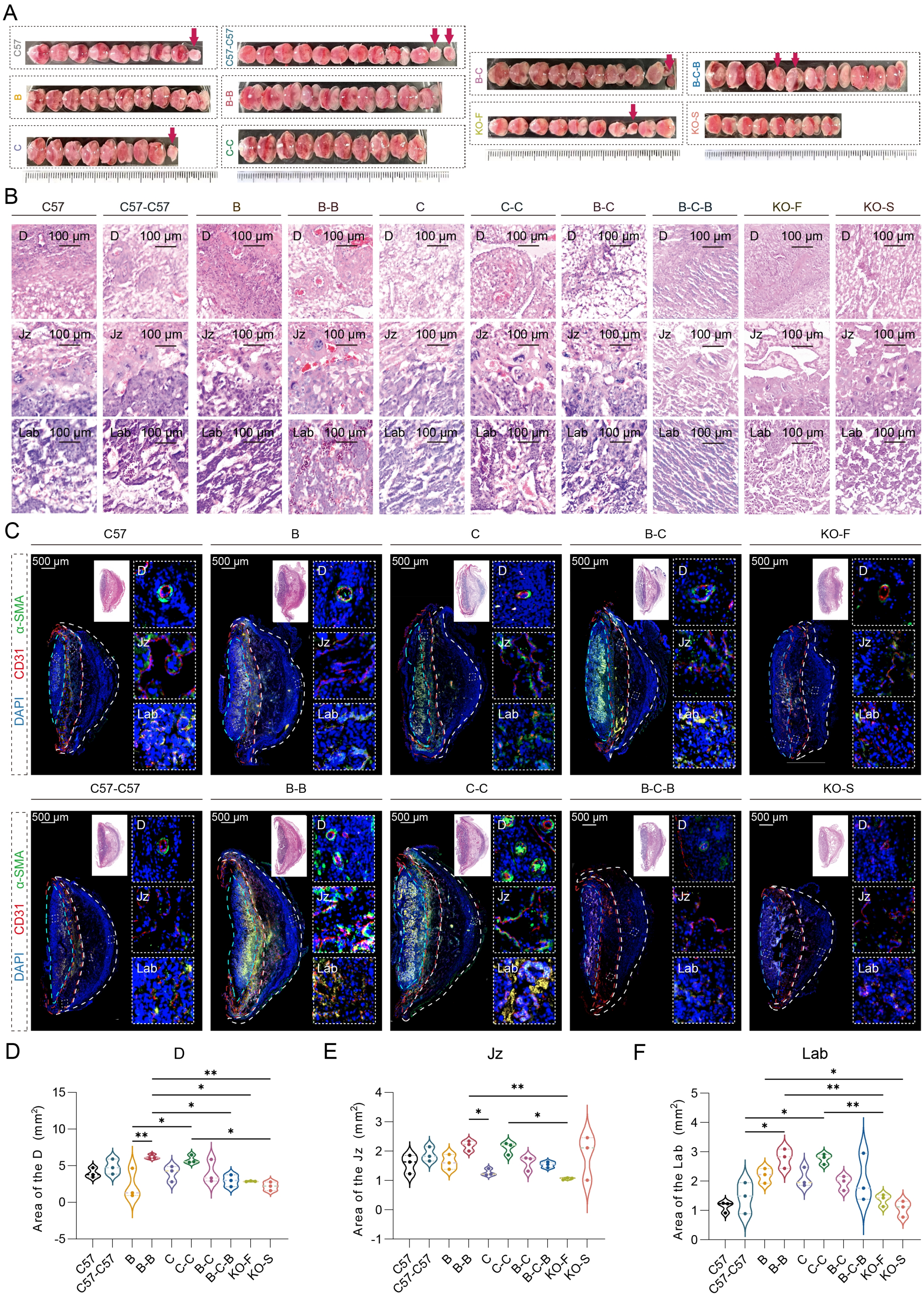

6

7 **Supplemental Figure 2.** Comparison of fetal and placental development in syngeneic
8 and allogeneic mouse pregnancies. (A) Representative images of fetuses at gestational
9 day (GD) 11.5. (B) Hematoxylin and eosin staining of the decidua (D), junctional
10 zone (Jz), and labyrinth (Lab) in placentas at GD11.5. Scale bar, 100 μm.

(C) Immunofluorescence staining of placental vasculature ($CD31^+$, red; α-SMA^+, green; DAPI, blue) at GD11.5. Scale bar, 500 μm. (D–F) Quantification of the area of the D (D), Jz (E), and Lab (F) at GD11.5. For quantification in D–F, three mice were randomly selected per group, with one placenta was collected from each mouse for measurements. Data in D–F are presented as mean ± SEM. Statistical differences were determined by one-way ANOVA in D–F; *$p < 0.05$, **$p < 0.01$.

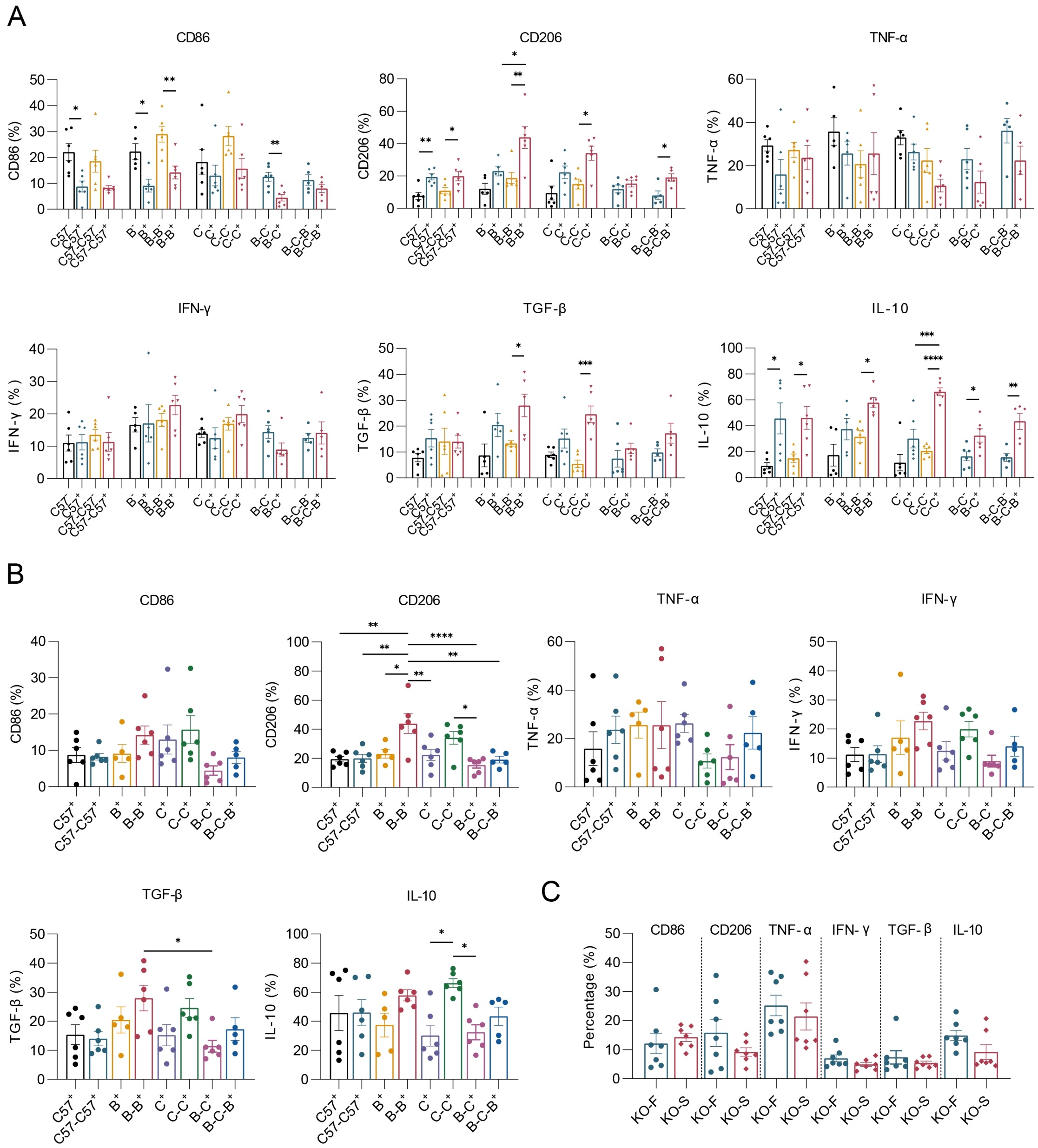

Supplemental Figure 3. Phenotypic and functional characteristics of PIR-B$^+$ macrophages in syngeneic and allogeneic mouse pregnancies. (A) Phenotypic and functional analysis of PIR-B$^-$ and PIR-B$^+$ uterine macrophages at GD11.5 (C57$^-$, PIR-B$^-$ uterine macrophages from C57 group; C57$^+$, PIR-B$^+$ uterine macrophages from C57 group; naming convention applies to other groups). (B) Phenotypic and functional analysis of PIR-B$^+$ uterine macrophages at GD11.5. (C) Phenotypic and functional analysis of uterine macrophages at GD11.5 (KO-F, macrophages from *Pirb*$^{-/-}$ mice in first pregnancy; KO-S, macrophages from *Pirb*$^{-/-}$ mice in second pregnancy). Data are mean ± SEM. Statistical differences were determined by

one-way ANOVA in A, B, and unpaired two-tailed t-test in C; *$p < 0.05$, **$p < 0.01$, ***$p < 0.001$, ****$p < 0.0001$.

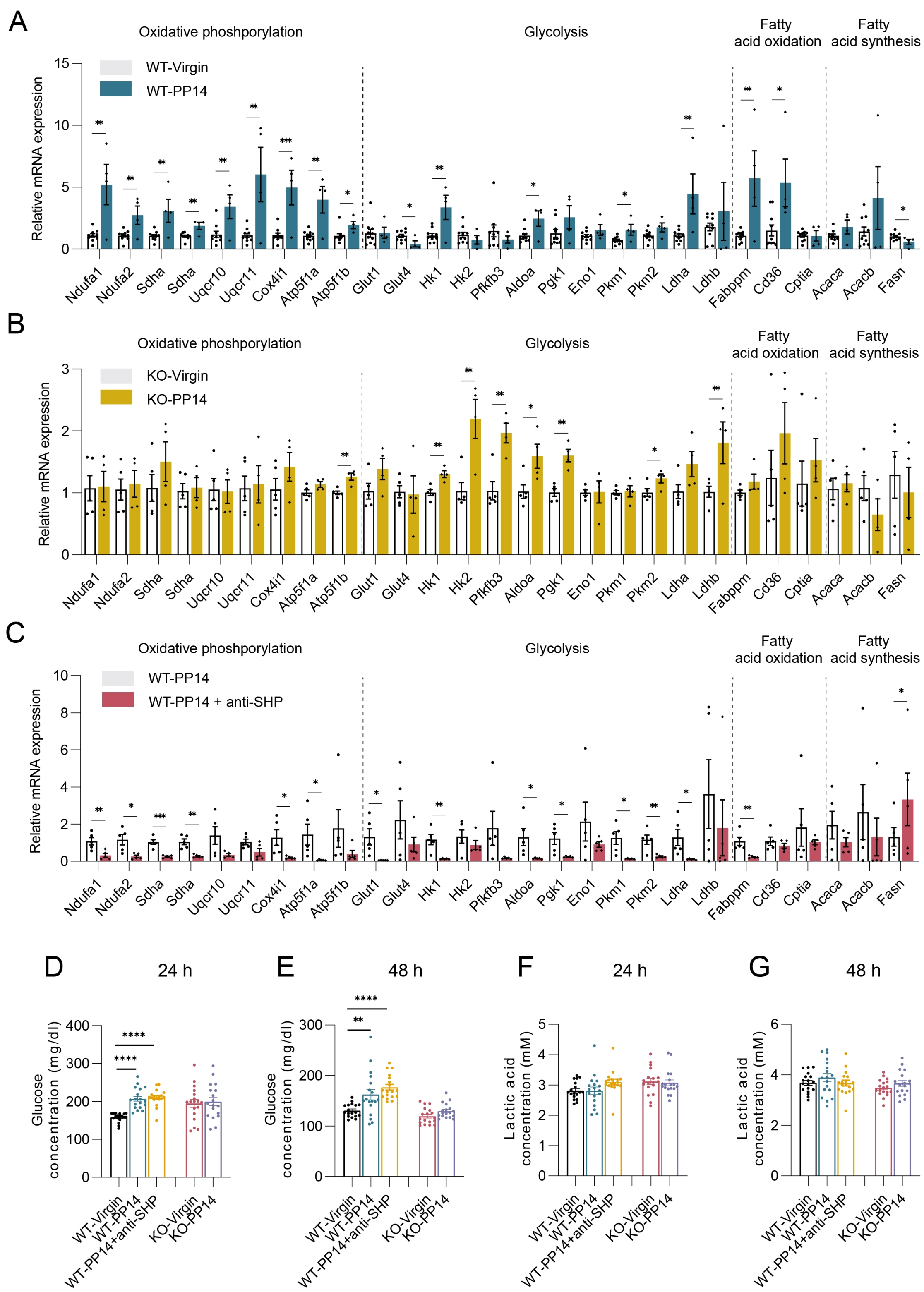

Supplemental Figure 4. Metabolic characterization of bone marrow-derived macrophages (BMDMs) under regulation by the PIR-B-SHP axis. (A–C) mRNA levels of metabolism-related genes in BMDMs from the following groups: (A) WT-Virgin vs WT-PP14, (B) KO-Virgin vs KO-PP14, (C) WT-PP14 vs WT-PP14 +

anti-SHP. (Abbreviations: WT-Virgin, wild-type virgin mice; WT-PP14, wild-type mice at postpartum day 14; WT-PP14 + anti-SHP, wild-type mice at postpartum day 14 with SHP blockade during pregnancy; KO-Virgin, *Pirb*$^{-/-}$ virgin mice; KO-PP14, *Pirb*$^{-/-}$ mice at postpartum day 14.) (D and E) Glucose levels in culture supernatants at (D) 24 h and (E) 48 h. (F and G) Lactic acid levels in culture supernatants at (F) 24 h and (G) 48 h. Data are mean ± SEM. Statistical differences were determined by unpaired two-tailed t-test in A-G and by one-way ANOVA in D-G; *$p < 0.05$, **$p < 0.01$, ***$p < 0.001$, ****$p < 0.0001$.

Supplementary Tables

Supplementary Table 1. Detailed characteristics of the first-trimester pregnant cohort.

Decidua samples related to Figure 1B, D, E, F, H, J; Figure S1A

Gravidity	Parity	Gestational days	Age	LILRB3$^+$/CD14$^+$(%)
1	0	48	28	8.64
1	0	28	27	5.3
1	0	72	23	8.48
1	0	72	30	6.65
1	0	48	23	18.41
1	0	47	18	30.2
1	0	50	26	9.77
1	0	28	20	14.26
1	0	53	23	10.91
1	0	38	29	7.38
1	0	47	26	6.89
1	0	72	18	12.22
1	0	59	29	19.07
1	0	57	17	34.16
1	0	37	24	6.35
1	0	52	25	12.03
1	0	56	19	35.44
1	0	45	23	17.64
1	0	69	25	18.18
1	0	47	22	9.37
1	0	38	20	10.77
1	0	43	19	7.76
1	0	42	33	10.28
1	0	28	25	9.43
1	0	28	20	10.71
1	0	28	23	17.27
2	1	52	35	3.83
4	2	32	37	12.14
3	1	84	23	53.81
2	1	44	24	48.41
2	1	47	31	36.33
2	1	49	33	27.82
4	2	77	28	59.29
2	1	50	32	23.08
3	1	84	31	40.25
3	2	43	33	26.25

2	1	40	31	42.37
3	0	75	25	32.45
4	2	64	32	35.99
2	1	45	32	17.13
3	2	70	24	15.92
3	1	63	26	14.12
2	1	73	31	12.05
3	1	49	19	9.66
5	2	48	20	27.2
2	0	52	21	11.63
6	1	61	36	37.92
2	1	63	33	46.75
3	1	58	36	29.64
4	1	59	30	18.87
4	2	80	26	67.5
3	1	70	24	26.23
5	0	54	23	40.91
2	1	71	35	51.49
2	0	50	22	28.54
2	0	50	35	17.24
2	1	43	32	34.38
3	1	70	32	48.1
2	1	60	36	25.3
2	1	47	32	15.75
3	1	57	39	34.27
2	0	40	24	19.57
6	2	42	42	41.44
2	0	48	20	20.54
2	0	77	20	34.72
2	1	77	26	26.54
2	1	42	34	25.4
3	1	52	35	8.67
2	1	28	33	9.93
5	2	42	34	19.42
5	2	49	36	44.97
3	1	45	35	4.39
3	1	42	29	11.03
3	2	40	38	18.81
4	2	56	37	16.86
5	4	56	35	30.17
4	1	44	33	22.43
2	1	55	30	18.01
4	1	55	22	22.82
2	1	48	26	11.48

3	0	28	29	16.53
4	1	38	27	24.86
2	1	36	30	21.38
2	1	28	22	17.1

Supplementary Table 2. Detailed characteristics of the third-trimester pregnant cohort.

Decidua samples related to Figure 1C, D, E, G, I, K; Figure S1B

Gravidity	Parity	Gestational days	Age	$LILRB3^{+}/CD14^{+}$(%)
1	1	271	22	36.04
1	1	279	24	21.1
1	1	285	26	29.74
1	1	278	20	41.84
1	1	284	26	23.24
1	1	280	34	12.07
1	1	286	22	19.11
1	1	280	20	12.27
1	1	270	21	31.73
1	1	262	24	32.57
2	2	259	31	44.87
3	2	263	27	27.66
2	2	272	30	50.92
3	2	268	31	46.42
3	3	270	31	58.2
3	2	275	24	40.42
2	2	263	28	35.46
5	3	276	35	50.24
2	1	266	24	58.92
3	3	268	28	21.31
6	2	272	34	50.32
2	2	280	35	38.64
2	1	271	32	9.73
2	2	261	39	5.22
2	2	275	23	15.44
2	2	277	33	51.3
3	2	272	37	50.25
2	2	274	26	58.33
2	2	273	28	56.41
2	1	274	26	59.19
2	1	276	30	27.22
3	2	269	30	34.54
2	2	266	32	55.8
2	2	273	26	26.1
4	3	273	32	38.85
3	1	272	27	26.83
5	1	273	26	46.97

Supplementary Table 3. Decidua samples used for RNA-seq.

Decidua samples related to Figure 4A-J

	Gravidity	Parity	Gestational days	Age
Primigravida				
Sample 1	1	0	47	23
Sample 2	1	0	51	24
Multigravida				
Sample 1	2	1	44	29
Sample 2	4	1	52	30

Supplementary Table 4. Decidua samples used for phenotype and cytokine detection.

Decidua samples related to Figure 4K

	Gravidity	Parity	Gestational days	Age
Primigravida				
Sample 1	1	0	60	21
Sample 2	1	0	56	28
Sample 3	1	0	56	18
Sample 4	1	0	53	26
Sample 5	1	0	46	25
Sample 6	1	0	58	33
Sample 7	1	0	47	20
Sample 8	1	0	51	24
Multigravida				
Sample 1	5	2	57	31
Sample 2	2	1	52	30
Sample 3	3	0	61	23
Sample 4	3	1	65	29
Sample 5	4	2	49	34
Sample 6	3	0	38	20
Sample 7	5	2	59	35
Sample 8	5	2	58	38
Sample 9	3	1	60	30
Sample 10	6	2	55	34
Sample 11	3	1	55	30
Sample 12	4	1	48	30
Sample 13	4	1	80	37
Sample 14	4	1	44	31

Supplementary Table 5. Antibody used for flow cytometry.

Antibody	Fluorochrome	Source	Identifier
Human			
CD14	FITC	Biolegend	325604
LILRB1	Percp-cy5.5	Biolegend	333714
LILRB2	APC	Biolegend	338708
LILRB3	PE	Biolegend	337704
CD80	PE-cy7	Biolegend	305218
CD86	PE-cy7	Biolegend	25-0869-42
CD206	PE-cy7	Biolegend	25-2069-42
CD209	Percp-cy5.5	Biolegend	330110
TNF-α	APC	Biolegend	502912
IFN-γ	APC	Biolegend	502512
TGF-β	APC	Biolegend	300006
IL-10	APC	Biolegend	501409
Mice			
CD45	APC-cy7	Biolegend	103116
F4/80	FITC	Biolegend	123108
PIR-B	APC	RD	FAB2754A
PIR-B	PE	RD	FAB2754P
CD86	Percp	Biolegend	105026
CD206	Percp-cy5.5	Biolegend	141716
TNF-α	PE-cy7	Biolegend	506324
IFN-γ	PE	Biolegend	505808
TGF-β	Percp-cy5.5	Biolegend	141410
IL-10	Percp-cy5.5	Biolegend	505027

Supplementary Table 6. Antibody used for immunohistochemistry (IHC), immunofluorescence (IF) and western blot (WB).

Antibody	Source	Identifier
Human		
LILRB3	Abcam	ab271287
LILRB3	Invitrogen	PA5-97926
CD68	Santa	sc-20060
Mice		
CD31	Proteintech	28083-1-AP
α-SMA	Abcam	ab21027
NDUFB8	Proteintech	14794-1-AP
SDHA	Proteintech	14865-1-AP
UQCRC2	Proteintech	14742-1-AP
COXIV	Proteintech	11242-1-AP
ATP5F1	Proteintech	15999-1-AP
FASN	Abcam	ab128870
β-tubulin	Servicebio	GB152667-100

Supplementary Table 7. Primers used in this study.

PCR	Forward primer (5' to 3')	Reverse primer (5' to 3')
Mice		
Ndufa1	GGGGCAAGGAAAAACGAGTTG	ACATAGTAGCGATTGACTCCAGA
Ndufa2	CTGGGGTTGCGTGAGATTC	CCGTTGCACGATGAAATCCC
Sdha	TGGACATCAAGACTGGCAAGGTTAC	GTAGGAGCGGATAGCAGGAGGTAC
Sdhb	AGTGCGGACCTATGGTGTTG	AGACTTTGCTGAGGTCCGTG
Uqcr10	CGCAGAACTTCCACCTTTGC	CCCTCGTTGATGTGCTCGTA
Uqcr11	GGACTGGGTGCCTTACATCAACG	GTGTCCAGCTTCCTCAGTGTCTTC
Cox4i1	TGTACCGCATCCAGTTTAACGAGAG	CGCAGTGAAGCCAATGAAGAACATG
Atp5f1a	GTCGCTTACCGCCAGATGTCTC	AAAGAGCCACCACCAAAGGAATCG
Atp5f1b	TCCTGCCAGAGACTATGCG	GATGACTGCCACGATTCGC
Glut1	TGTGCTTCCAGTATGTGGAGC	CTTGTCACTTTGGCTGGCAC
Glut4	ATGTTGCGGATGCTATGGGTC	CTGGGTTTCACCTCCTGCTC
Hk1	TCCATCCACACTTCTCCAGAATC	GATCCTGGCTCTTAGGCGTTC
Hk2	AGAACATGGCGAGTTCCTGG	TGTGGTCAAACAGCTGGGTT
Pfkfb3	TGTCCAGCAGAGGCAAGAAG	TCTCGTTGAGTGCCTTCCAC
Aldoa	ACTTGAAGCGCTGCCAGTA	AGGGGGCACTTGTTGATAGC
Pgk1	AGCTACTGTGGCCTCTGGTA	GAGTGACTTGGTTCCCCTGG
Eno1	TCATTGGCATGGATGTGGCT	CGCTTAGGGTTGGTCACTGT
Pkm1	GTCTGGAGAAACAGCCAAGG	TCTTCAAACAGCAGACGGTG
Pkm2	GTCTGGAGAAACAGCCAAGG	CGGAGTTCCTCGAATAGCTG
Ldha	CAAAGACTACTGTGTAACTGCGA	TGGACTGTACTTGACAATGTTGG
Ldhb	TTCCTCCAGACTCCGAAAATTG	GTTTCCAGGTGACGTAAGTCAG
Fabppm	GCGTTACCGAAGCCTTCAAGA	GGAGCACGTAAGGTTTTCCGT
Cd36	GGCCAAGCTATTGCGACATG	AGGCATTGGCTGGAAGAACA
Cpti1	AGCTAAAATCCTGGTGGGCC	GCAGAGCAGAGGGGAATTGT
Acaca	CTCCCGATTCATAATTGGGTCTG	TCGACCTTGTTTTACTAGGTGC
Acacb	AGGTTCCAGATGCTAATGGGT	GCCCAGGATAAAGCTGGTCAT
Fasn	CAAGTGTCCACCAACAAGCG	CCCAAGGAGTGCCCAATGAT
β-actin	TGCGTGACATCAAAGAGAAG	TCCATACCCAAGAAGGAAGG
Genotyping primers		
PCR Primers1	ATGCCACTAGGTGGTAAGTATCAG	GTTCTAAGTCCAAAAGATTTGCCTG
PCR Primers2	AAATCACATACCTTTCTCTCACCCTC	GTTCTAAGTCCAAAAGATTTGCCTG
